# Systematic Assessment of Deep Learning-Based Predictors of Fragmentation Intensity Profiles

**DOI:** 10.1021/acs.jproteome.3c00857

**Published:** 2024-05-10

**Authors:** Mehdi B. Hamaneh, Aleksey Y. Ogurtsov, Oleg I. Obolensky, Yi-Kuo Yu

**Affiliations:** National Center for Biotechnology Information, National Library of Medicine, National Institutes of Health, Bethesda, Maryland 20894, United States

**Keywords:** mass spectrometry, spectrum prediction, deep learning

## Abstract

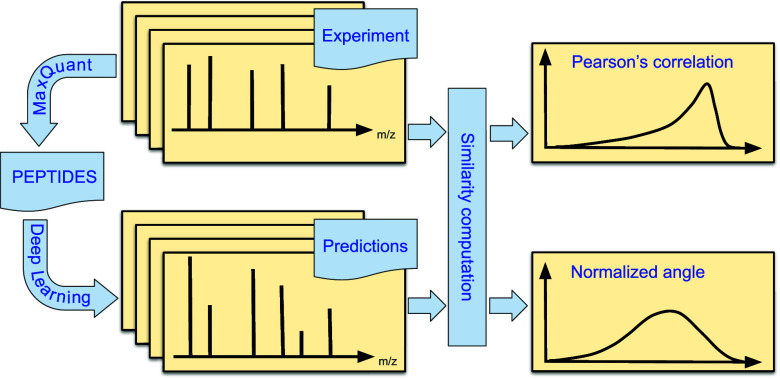

In recent years, several deep learning-based methods have been proposed for predicting peptide fragment intensities. This study aims to provide a comprehensive assessment of six such methods, namely Prosit, DeepMass:Prism, pDeep3, AlphaPeptDeep, Prosit Transformer, and the method proposed by Guan et al. To this end, we evaluated the accuracy of the predicted intensity profiles for close to 1.7 million precursors (including both tryptic and HLA peptides) corresponding to more than 18 million experimental spectra procured from 40 independent submissions to the PRIDE repository that were acquired for different species using a variety of instruments and different dissociation types/energies. Specifically, for each method, distributions of similarity (measured by Pearson’s correlation and normalized angle) between the predicted and the corresponding experimental *b* and *y* fragment intensities were generated. These distributions were used to ascertain the prediction accuracy and rank the prediction methods for particular types of experimental conditions. The effect of variables like precursor charge, length, and collision energy on the prediction accuracy was also investigated. In addition to prediction accuracy, the methods were evaluated in terms of prediction speed. The systematic assessment of these six methods may help in choosing the right method for MS/MS spectra prediction for particular needs.

## Introduction

The sensitivity and specificity of mass spectrometry-based peptide, protein, and microorganism identification can potentially be aided by employing reliable data for peptide fragmentation profiles. These profiles can be made available not only through physical modeling of peptide fragmentation process^1−5^ or through building of extensive spectral libraries,^6−10^ but also by employing deep learning algorithms to predict fragmentation profiles for each precursor on-the-fly. This can be especially useful for data-independent acquisition (DIA) proteomics. The current state of development of neural-network-based methods already allows for precise predictions of fragmentation profiles.

For example, Xu et al.^11^ compared three deep learning-based approaches (Prosit,^12^ pDeep2,^13^ and Guan’s method^14^) with MS/MSPIP^15^ (a random forest regression learning algorithm) and showed that the deep learning-based methods were superior and that these methods achieved high accuracy. Since the benchmarking study of Xu et al., a few other deep learning-based methods have been developed, some of which utilize the state-of-the-art Transformer architecture. A systematic assessment of, and comparison among, these recently proposed methods is lacking. This study aims to provide a detailed independent evaluation and comparison of these methods as well as (for completeness) older deep learning-based approaches.

To make a comprehensive assessment, we conducted a literature search to find MS/MS spectrum prediction methods. The search was not limited to a specific publication time period, but we selected only methods that (1) utilize deep learning, (2) can be applied to a wide range of data obtained using various instruments, and (3) accept as input only peptide sequence (possibly with some post-translational modifications, PTMs) and charge and optionally MS experiment-related parameters such as collision energy. Based on these criteria, in this study, we included six MS/MS spectrum prediction methods, namely AlphaPeptDeep^16^ (PeptDeep for short), Prosit Transformer,^17^ DeepMass:Prism^18^ (Prism for short), pDeep3,^19^ Prosit,^12^ and the method proposed by Guan et al.^14^ On the other hand, we excluded MS/MSCNN^20^ and DeepDIA^21^ because the former requires other types of input (such as peptide hydrophobicity), and the latter is recommended to be retrained for use in each lab, i.e. it does not satisfy our criterion (2) above. In other words, despite its reported high prediction accuracy, DeepDIA cannot be fairly compared with other methods because it is supposed to be retrained for each instrument in each lab. We also excluded PredFull^22^ which was developed to predict the full MS/MS spectra. Since other methods focus on training their networks to accurately predict intensities of *b* and *y* fragments, a comparison with less focused predictions of PredFull would have been unfair to PredFull. Of note, pDeep3 and PeptDeep are both based on pDeep2,^13^ which in turn is based on pDeep.^23^ Thus, we did not include pDeep and pDeep2 in our study.

The six included methods employ neural networks designed to handle sequential data and thus are suitable for predicting spectra from peptide sequence. Specifically, the methods use bidirectional long short-term memory (pDeep3, Prism, and Guan’s method), gated recurrent memory units (Prosit), or Transformers (Prosit Transformer and PeptDeep). The network architecture and other details about the included methods have been discussed in their respective publications and so are not repeated here.

To evaluate the six included methods, we used data from 40 independent submissions to the PRIDE repository, including 30 projects containing tryptic and 10 projects containing human leukocyte antigen (HLA) peptides, which are typically nontryptic. The 40 PRIDE projects comprise experimental spectra for more than 1.7 million precursors that were acquired from different species using different instruments, dissociation types, and collision energies. Here, for tryptic peptides, we investigate the impact of several variables (including instrument, dissociation type, methionine oxidation, collision energy, peptide length, and precursor charge) on the MS/MS spectrum prediction accuracy of each method (using intensities of singly- and doubly-charged *b* and *y* fragments). Also, we compare the performance of the methods when they are applied to HLA peptides. Additionally, the methods are also compared in terms of the prediction speed.

## Methods

### Data: Tryptic

The experimental data in Thermo Scientific’s .raw file format were downloaded from 30 different submissions to the publicly accessible PRIDE repository maintained by the European Bioinformatics Institute.^24^ These contributions were chosen randomly from those uploaded within the past few years and large enough to contain at least 10 .raw files. Since most prediction methods could not handle tandem mass tag labeling (TMT), this type of data was excluded. These requirements allowed us to operate with spectra measured at higher resolutions and to set aside some data for subsequent fine-tuning of neural network parameters or normalized collision energy (NCE) calibration for the methods that allowed such additional steps. We also tried to maintain a balance between different types of dissociation and different instruments and to have a representation of different organisms. The instruments covered in this study are Q Exactive (QE; including QE HF and QE Plus), Fusion, Fusion Lumos (which we henceforth refer to as Lumos), and Velos. We included spectra obtained using collision-induced dissociation (CID) and higher-energy collisional dissociation (HCD), but not electron transfer dissociation (ETD) as most evaluated predictors are not able to make predictions for this type of data. Overall, we believe that the collected data adequately capture the current usage of mass spectrometry in biomedical applications. We used fasta files from representative proteomes downloaded from https://www.uniprot.org/proteomes,^25^ unless the submission provided their own, specific for the project, fasta files. Some of the submissions contained more than one type of dissociation type and/or instrument and/or NCE. In such instances, we considered the spectra with the same combinations of such parameters, even within one PRIDE project, as separate spectral “datasets”. Taking this approach, we ended up with 36 datasets (see the “Preparing the Input” subsection for details) whose parameters are provided in Table 1.

**Table 1:** Datasets Used in Evaluation of Fragmentation Intensity Profiles

PRIDE Id	organism	dissociation	NCE	instrument	PSMs^a^	precursors
PXD008851	*Bos taurus* (bovine)	CID	35	Velos	313,634	6,267
PXD012025	*Drosophila melanogaster* (fruit fly)	HCD	27	QE	72,876	13,769
PXD013270	*Rattus norvegicus* (rat)	HCD	27	QE	225,809	42,419
PXD014374	*Bos taurus* (bovine)	HCD	28	QE	809,031	30,111
PXD014759	*Saccharomyces cerevisiae* (baker’s yeast)	CID	30	Fusion	260,212	24,579
PXD014759	*Saccharomyces cerevisiae* (baker’s yeast)	CID	35	Fusion	428,496	21,264
PXD015306	*Drosophila melanogaster* (fruit fly)	CID	35	QE	312,950	62,196
PXD015442	*Rattus norvegicus* (rat)	HCD	30	Lumos	1,290,673	13,714
PXD016688	*Homo sapiens* (human)	CID	29	Fusion	1,262,429	115,390
PXD016793	*Rattus norvegicus* (rat)	HCD	27	QE	1,060,883	102,638
PXD016986	*Riftia pachyptila* (tubeworm),	CID	35	Velos	2,284,782	66,820
	*Endoriftia persephone* (bacterium)					
PXD019296	*Rattus norvegicus* (rat)	HCD	27	QE	378,592	33,154
PXD020557	*Homo sapiens* (human)	HCD	27	QE	223,391	12,973
PXD020756	*Bos taurus* (bovine)	HCD	30	QE	127,252	7,144
PXD020859	*Rattus norvegicus* (rat)	HCD	30	QE	782,324	77,613
PXD020859	*Rattus norvegicus* (rat)	HCD	30	Fusion	55,215	12,510
PXD021588	*Homo sapiens* (human)	HCD	26	QE	992,521	64,646
PXD026323	*Saccharomyces cerevisiae* (baker’s yeast)	HCD	28	QE	43,315	13,411
PXD030971	*Staphylococcus aureus* (bacterium)	CID	35	Velos	817,158	41,921
PXD031222	*Proteus mirabilis* HI4320 (bacterium),	HCD	27	QE	679,732	39,887
	*Candida albicans* (yeast)					
PXD031222	*Proteus mirabilis* HI4320 (bacterium),	HCD	30	QE	706,277	40,019
	*Candida albicans* (yeast)					
PXD032834	*Plasmodium falciparum* (isolate 3d7) (malaria parasite)	HCD	25	QE	7,348	6,178
PXD032834	*Plasmodium falciparum* (isolate 3d7) (malaria parasite)	HCD	28	QE	37,558	23,323
PXD033348	*Saccharomyces cerevisiae* (baker’s yeast)	HCD	27	QE	132,912	13,976
PXD034156	*Mus musculus* (mouse)	HCD	27	QE	964,972	81,112
PXD035632	*Synechocystis* sp. PCC 6803 (bacterium)	HCD	26	QE	61,052	10,626
PXD035632	*Synechocystis* sp. PCC 6803 (bacterium)	HCD	27	QE	135,466	18,869
PXD035677	*Arabidopsis thaliana* (mouse-ear cress)	HCD	30	Lumos	168,653	31,242
PXD036475	*Escherichia coli* (bacterium)	HCD	35	Fusion	502,245	80,800
PXD036796	*Staphylococcus aureus* (bacterium)	HCD	28	QE	517,601	28,119
PXD040423	*Mus musculus* (mouse)	HCD	30	Lumos	434,351	62,067
PXD040724	*Caenorhabditis elegans* (nematode)	HCD	25	QE	118,429	26,340
PXD041442	*Mus musculus* (mouse)	HCD	27	QE	169,770	48,149
PXD042301	*Mus musculus* (mouse)	HCD	27	QE	210,729	32,741
PXD042322	*Trypanosoma brucei* (kinetoplastid)	CID	35	Fusion	41,009	13,075
PXD042322	*Trypanosoma brucei* (kinetoplastid)	HCD	30	Fusion	19,417	8,876
total					16,649,064	1,327,938

a Precursor spectrum matches, see section Workflow.

### Data: HLA

We searched the PRIDE repository to find projects containing HLA data to assess the performance of the methods in predicting the fragment intensity profiles of such peptides. Our search queries did not result in any PRIDE projects that utilized Fusion or Velos instruments and that we deemed suitable for inclusion in our analysis. In the end, we picked 10 PRIDE projects with data acquired using QE and Lumos instruments to assess the performance of the methods. Some of these projects contain tryptic as well as HLA data. In these cases, the .raw files containing tryptic data were excluded from further analysis. As described above, if a PRIDE project contained data obtained using multiple dissociation type/instrument/NCE, the project was partitioned (see the “Preparing the Input” subsection for details) into separate “datasets” containing data with the same type/instrument/NCE. This process resulted in 14 HLA datasets whose parameters are provided in Table 2.

**Table 2:** Datasets Used in Evaluation of Fragmentation Intensity Profiles of HLA Peptides

PRIDE Id	HLA class	dissociation	NCE	instrument	PSMs	precursors
PXD004894	I and II	HCD	25	QE	63,358	23,803
PXD004894	I and II	HCD	27	QE	581,562	127,495
PXD006939	I and II	HCD	27	QE	600,372	176,878
PXD008500	I	HCD	25	QE	59,106	14,089
PXD009925	I	HCD	27	QE	96,646	42,031
PXD011628	I	CID	35	Lumos	151,396	19,769
PXD020079	I and II	HCD	27	QE	370,664	80,379
PXD020186	I and II	HCD	30	Lumos	45,260	10,384
PXD020186	I and II	CID	35	Lumos	37,687	8,097
PXD027182	I	CID	35	Lumos	94,318	11,883
PXD028985	I	HCD	27	QE	92,538	16,551
PXD037270	I	HCD	27	Lumos	75,426	30,941
PXD037270	I	HCD	30	Lumos	75,369	31,573
PXD037270	I	HCD	32	Lumos	217,984	42,458
total					2,561,686	636,331

### Assessed Methods

As mentioned previously, in this study we assess and compare six spectrum prediction methods that are PeptDeep,^16^ Prosit Transformer,^17^ Prism,^18^ pDeep3,^19^ Prosit,^12^ and the method proposed by Guan et al.^14^ The network types used by these methods, the ion types for which the intensities are predicted, the dissociation type (DT) that each method can handle, the required input, and the limitations (the range of accepted precursor charge *Z* and length *N*) of each approach are given in Table 3.

**Table 3:** Methods Assessed in This Study

method	network	DT	ions^a^	input^b^	limitations	TMT^c^
Guan et al.	BiLSTM^d^	HCD	*b*, *y* (+, ++)	S, *Z*	*N* ≤ 40, *Z* ≤ 6	no
Prosit	BDN + GRU + AL^e^	HCD/CID	*b*, *y* (+, ++, +++)	S, *Z*, NCE	*N* ≤ 30, *Z* ≤ 6	yes
Prism	BiLSTM	HCD/CID	*b*, *y*, NL (+)	S, *Z*, DT, MAT	*Z* ≤ 7	no
pDeep3	BiLSTM	HCD/ETD	*b*, *y*, *c*, *z*, NL (+, ++)	S, *Z*, NCE, DT, INS	*Z* ≤ 7	no
Prosit T^f^	transformer	HCD	*b*, *y* (+, ++, +++)	S, *Z*, NCE	*N* ≤ 30, *Z* ≤ 6	no
PeptDeep	transformer	HCD	*b*, *y*, NL (+, ++)	S, *Z*		no

a Singly-, doubly-, and triply charged ions are respectively denoted by + , ++, and +++. NL denotes neutral loss.

b S: precursor amino acids sequence, *Z*: precursor charge, NCE: normalized collision energy, DT: dissociation type, MAT: mass analyzer type, INS: instrument.

c Yes (No): The method is (not) capable of analyzing TMT data.

d BiLSTM: bidirectional long short-term memory.

e BDN:bidirectional recurrent neural network; GRU: gated recurrent memory units; AL: attention layer.

f Prosit T denotes Prosit Transformer.

### Workflow

**Figure 1:**
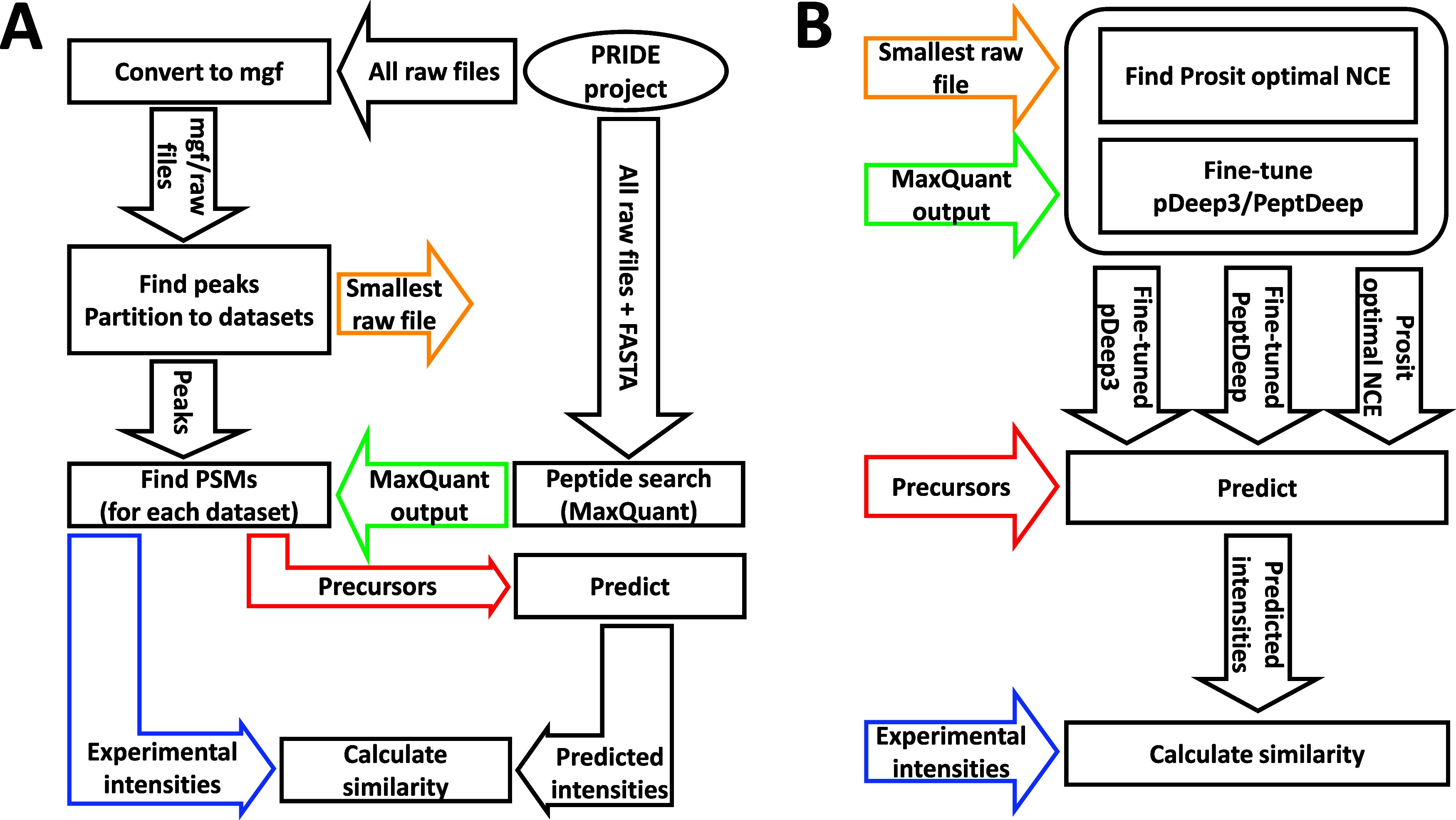
Workflow of the study. Each rectangle in the figure represents an action, whereas each arrow shows an input/output for/from an action. (A) Workflow with no calibration/fine-tuning. The “MaxQuant output” is the “msms.txt” file. A “dataset” refers to spectra with the same instrument/energy/dissociation/mass analyzer combination. For each dataset the smallest .raw file was set aside for use only in the NCE-calibration/fine-tuning process (see panel B). Inputs/outputs used for NCE-calibration/fine-tuning are color-coded for clarity. (B) Workflow for NCE-calibration of Prosit, and fine-tuning of pDeep3 and PeptDeep. The color-coded inputs are from panel A. MaxQuant output is the “msms.txt”, for Prosit and pDeep3, or “evidence.txt” file for PeptDeep.

As described in the following subsections, the data were processed to identify peptide-spectrum matches or, more precisely, precursor-spectrum matches (PSMs), where by “precursor” we mean the peptide sequence and its charge. We then created the inputs for the prediction methods and assessed the prediction accuracy of each method. The workflow is summarized graphically in Figure 1.

#### Extracting the Spectra

First, an in-house program was employed to convert the .raw files in each of the 40 PRIDE projects to the MGF format. Then each MGF file was used to extract the spectra corresponding to the scan numbers. Additionally, the instrument (INS) used for performing the experiment, the dissociation types (DT), the mass analyzer types (MAT), the precursor charges (*Z*), and the normalized collision energies (NCE) for different spectra were retrieved from each .raw file and were saved for subsequent use. Since some of the evaluated methods can be fine-tuned and/or NCE-calibrated for improved performance (see the “Fine Tuning and NCE Calibration” subsection), the smallest .raw file in each dataset was set aside for fine-tuning/NCE-calibration.

#### Identifying PSMs

We used MaxQuant^26^ version 2.1.3.0 to find PSMs. For tryptic data, we ran MaxQuant using the default parameters. However, two changes were made when running MaxQuant for HLA data: enzyme specificity was set to “unspecific” and protein false discovery rate was chosen to be 1. For all PRIDE projects, except for PXD020859, MaxQuant was run for all of the .raw files in the PRIDE project (including the ones set aside for fine-tuning) at the same time. Since experimental data in PXD020859 were obtained using two different instruments, MaxQuant was run separately for the .raw files in this PRIDE project based on the instrument used. MaxQuant was not able to read three .raw files from three PRIDE projects (PXD004894, PXD013270, and PXD014759) and so these three .raw files were excluded from all subsequent analyses.

The “msms.txt” file from MaxQuant output was utilized to identify the scan number corresponding to each S/*Z*/NCE/DT/INS/MAT combination, where S denotes the sequence. Peptides with PTMs were excluded from further analysis except for the ones with only methionine oxidation and/or Cysteine carbamidomethylation (fixed modification). This is because some of the methods assessed in this study do not accept any other modifications. Any peptide containing U or O amino acids was also excluded. We then matched the scan numbers from the .raw files to those obtained from MaxQuant to find the PSMs, i.e., the spectra corresponding to each S/*Z*/NCE/DT/INS/MAT combination. Since most of the methods assessed in this study (see the “Assessed Methods” subsection) predict only intensities associated with singly- and doubly-charged *b* and *y* ions, only experimental peaks corresponding to these ions were considered. To find the intensity of these experimental peaks, the theoretical *m*/*z* values were calculated and matched to the experimental ones. (Here *m* and *z* denote fragment mass and charge, respectively.) For consistency, we used the same peak-matching tolerances used in MaxQuant, i.e., 20 ppm for the Fourier Transform Ion Cyclotron Resonance Mass (FTMS) and 0.5 Da for the Ion Trap Mobility Spectrometry technology (ITMS) peaks, respectively. If no matching experimental peak was found, then the corresponding experimental intensity was considered to be zero.

#### Preparing the Input

To better differentiate the effect of INS, NCE, and DT on the performance of each method, the spectra in each PRIDE project were partitioned into different “datasets”, each having the same NCE/DT/INS/MAT. (Most PRIDE projects included in this study contained only one dataset.) From each dataset, the smallest of the corresponding .raw files was set aside for fine-tuning/NCE-calibration (see the “Fine Tuning and NCE Calibration” subsection for details). The PSMs found by MaxQuant were then used to find the corresponding precursor for each spectrum in each of the remaining .raw files in the dataset. Similarly, for each dataset, each spectrum in the .raw file set aside for fine-tuning/NCE-calibration, was associated with the identified precursor by MaxQuant. It is worth noting that in some cases PSMs were identified by MaxQuant even though all corresponding experimental intensities were zero. We excluded such PSMs from our data. When considering intensity profiles accounting for only singly-charged fragments, we also excluded PSMs and predictions with zero intensities for all singly-charged fragments.

For each dataset, if more than one spectrum was associated with a precursor, the precursor was counted only once. In other words, for each dataset (excluding the .raw file set aside), we created a nonredundant set of precursors. In the case of PXD015306, the nonredundant set of precursors corresponding to 30.00/HCD/QE/FTMS and 35.00/CID/QE/ITMS contained, respectively, only 2 and 803 precursors. These two sets were excluded from further analyses because they had too few precursors. The latter dataset was the only CID data obtained using QE, so we thought the number of precursors was not large enough to make a reliable comparison between the methods for CID/QE data. Excluding these two sets left us with 36 tryptic (29 HCD and 7 CID), and 14 HLA (11 HCD and 3 CID) sets of precursors and the corresponding 50 (36 tryptic plus 14 HLA) spectral datasets (Tables 1 and 2) that were used for assessing the methods. Each set of precursors was written to a comma-separated (csv) file that was then used as input for the evaluated methods (one precursor per line with the corresponding S/*Z*/NCE/INS/DT/MAT).

Of note, we found the nonredundant set of precursors for each PRIDE project separately after the fine-tuning/NCE-calibration .raw file had been set aside. (Obviously, the same data should not be used for both fine-tuning and assessment; see the “Fine Tuning and NCE Calibration” subsection for details.) Therefore, a nonredundant (within a dataset) precursor may still be associated with spectra in other datasets (from the same or a different PRIDE project) and also with spectra in the file set aside for fine-tuning/NCE-calibration. It should be emphasized that in Tables 1 and 2 for each dataset the total number of PSMs is given (number of spectra in the dataset plus that of spectra in the .raw file set aside). Also, for each dataset, the total number of nonredundant precursors is reported in the tables (nonredundant precursors associated with the spectra in the dataset or the set aside .raw file).

#### Prediction

We used each of the six methods to predict the intensities of the singly- and doubly-charged (except for Prism, which predicts intensities of singly-charged fragments only) *b* and *y* ions for the aforementioned 50 (36 tryptic +14 HLA) nonredundant sets of precursors. Except for Prism and Prosit, the Python code of each method was downloaded and used for prediction. Prism and Prosit were run on the Google Cloud Machine Learning Engine (CMLE) and Prosit web server (https://www.proteomicsdb.org/prosit/), respectively. In both cases, the input was converted to the format required by the method, and the output was parsed to extract the predicted intensities. Of note, Prosit Transformer does not label the predicted intensities by ion name and charge. Instead, it outputs the *m*/*z* for each predicted intensity. In this case, we matched the theoretical and the output *m*/*z* values to find the intensity corresponding to each ion (using a tolerance of 20 ppm).

#### Similarity Measures

To evaluate and rank the performance of each method, we computed two previously used^12,17^ similarity measures between the vectors of predicted and experimental intensities, namely, Pearson’s correlation *r* and the normalized angle α. Normalized angle is defined as
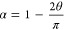
1where θ is the angle between the two vectors of predicted and experimental intensities. Normalized angle has the advantage of better distinguishing methods when similarity is high, because of its linear asymptotic behavior.^12^

Because the intensities are all positive, the normalized angle ranges between 0 and 1, with larger values indicating higher similarity. Since Prism does not predict the *b*^+2^ and *y*^+2^ intensities, we also computed the similarity measures for the intensity profiles of only singly-charged ions. The normalized angles and Pearson’s correlations were calculated for each PSM in a dataset and binned with the step of 0.001 resulting in discretized similarity distributions. The contribution from each PSM was weighted by the number of PSMs for the corresponding precursor so that each precursor from a dataset had an equal impact on the resulting distribution. The similarity distributions from different datasets were combined by adding corresponding bins from their distributions.

We used the obtained distributions to gauge the prediction quality for the six methods. In the ideal world, with perfect predictions and perfect reproducibility of fragmentation patterns in different experiments, the distributions would have only one nonzero component at the bin centered at 1. However, in the real world, not only the intensity predictions are not perfect but also the spectra of the same precursor, even in the same experiment, may differ. Therefore, the goodness of a predictor should be characterized not just by the closeness of its distribution to 1 but also by the distance between its distribution and the distribution of similarity between the experimental spectra corresponding to the same precursor.

In order to characterize the latter, for each precursor with more than one PSM, we introduced a reference experimental profile that was obtained by averaging the *L*^2^-normalized experimental intensity vectors. We then calculated the similarity distributions between these reference profiles and their corresponding experimental spectra in the same way as we computed the similarity distributions of the predicting methods.

The simplest way to quantify the similarity between two distributions is to compare their medians. However, one can employ more advanced measures for ranking and comparing distributions. For our analysis we chose the Wasserstein’s distance *W*_1_, also known as Kantorovich–Rubinstein metric or the earth mover’s distance.^27^ This metric satisfies the mathematical axioms of a distance and provides a more integral characterization of the difference between two normalized distributions. It basically measures how far intensity from one distribution should be moved so the second distribution is obtained:
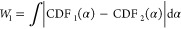
2where CDF are the cumulative distribution functions.

#### Fine Tuning and NCE Calibration

pDeep3 and PeptDeep both provide the user with the opportunity to fine-tune their model for usage in cases for which the model was not trained (e.g., CID data). Instead of training the model from scratch, fine-tuning uses a small number of PSMs and is run for only a few epochs, so it is fast to run and does not require a large amount of data. The PSMs required for fine-tuning are obtained from a .raw file and a “PSM file” that is a file that relates the sequences with their corresponding spectra (one of the output files of MaxQuant). As mentioned previously, from each dataset we set aside one .raw file to be used for fine-tuning of pDeep3 and PeptDeep. For pDeep3, the input PSM file was generated from MaxQuant “msms.txt”. The .raw and PSM files were then used to create a “.psmlabel” file using the psmLabel.exe command of pDeep3. Fine-tuning pDeep3 was then performed using the .psmlabel file as input. PeptDeep accepts different input formats for the spectra and PSM files for fine-tuning. The spectra file in the MGF format and the “evidence.txt” file from the MaxQuant output were used to fine-tune PeptDeep. The default parameters were used for fine-tuning, except that, for consistency, we used a peak matching tolerance of 0.5 Da for ITMS data. Also, the number of epochs for fine-tuning pDeep3 was set to 10, because this number was used for fine-tuning pDeep3 for CID data.^19^ Although the purpose of fine-tuning of pDeep3 and PeptDeep is to apply these methods to situations for which the methods have not been trained (CID data), we also used fine tuning for HCD data to investigate its effect on the performance of these methods when applied to this type of data. We refer to the fine-tuned versions of these two methods as pDeep3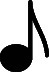 and PeptDeep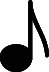.

As a part of fine-tuning, by default, pDeep3 also conducts a grid search for the pair of NCE and INS that gives the best results for the spectra file used for fine-tuning. In other words, the model is run for various pairs of (NCE, INS) and the best-performing pair is then identified. PeptDeep also provides the option for such a grid search but does not perform it by default during fine-tuning, so the grid search was not performed for PeptDeep.

The fine-tuning feature is not available in Prosit but it can be NCE-calibrated, which is recommended by Prosit developers for HCD data. By running the method (on a relatively small number of PSMs) using different values of NCE, the calibration finds the best-performing value of NCE. We used the same .raw file set aside for fine-tuning pDeep3 and PeptDeep for NCE-calibration of Prosit, which was performed using Prosit web server with the .raw and “msms.txt” files as input. Once the best value for NCE was obtained, Prosit input file was modified by changing the NCE to the value found during the calibration, and Prosit was run again using the modified input file. For NCE-calibration, Prosit requires the size of the .raw file to be smaller than 2 GB. In the case of PXD035632 all .raw files with NCE = 27 were larger than 2 GB, so NCE-calibration was not performed in this case. For a fair comparison, fine-tuning of pDeep3 and PeptDeep was not performed for this dataset either. Also, in the PRIDE project PXD042322, containing both 35.00/CID/Fusion/ITMS and 30.00/HCD/Fusion/ITMS datasets, there was no .raw file with only 30.00/HCD/Fusion/ITMS spectra (spectra with 30.00/HCD/Fusion/ITMS appeared in .raw files that contained the other combination as well). Therefore, no fine-tuning/NCE-calibration was performed for this dataset. In other words, 2 out of the 29 tryptic HCD datasets were excluded when evaluating Prosit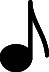 (the NCE-calibrated Prosit), pDeep3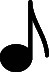, and PeptDeep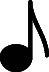. Additionally, when assessing these (fine-tuned/NCE-calibrate) methods, any precursor used for fine-tuning/NCE-calibration (precursors that were shared between the .raw file set aside for fine-tuning and other .raw files in the dataset) was excluded.

Of note, methods that do not offer the fine-tuning/NCE-calibration feature may still be trained from scratch to be applied to situations for which they have not been trained. However, our goal was to compare the performance of the methods in their current form, so we did not retrain any of the methods. For this reason, some of the methods are assessed for specific situations. Namely, Prosit Transformer and Guan’s method were assessed on HCD data only. On the other hand, we did fine-tune pDeep3/PeptDeep and calibrated Prosit, because fine-tuning/NCE-calibration is a feature of these methods and can be done without requiring a large amount of data and time.

## Results and Discussion

For each precursor in each of the 50 nonredundant sets of precursors (see Methods), the *b* and *y* ion intensities were predicted using the six evaluated methods as well as pDeep3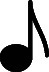, PeptDeep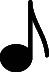, and Prosit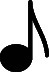 (the fine-tuned/NCE-calibrated versions of the corresponding methods). Some precursors cannot be processed by all evaluated methods. This is because of the various limitations that different methods have (Table 3). Hence, from each nonredundant set of precursors, we used only the subset that could be processed by all methods, namely, the precursors with *N* ≤ 30 and *Z* ≤ 6. In rare (less than 1% of) cases, Prosit did not output prediction for long (but still shorter than 30) peptides with PTMs. In the subsequent analyses, these precursors were excluded for Prosit but included for other methods. Given the rarity of such cases, our results should not be significantly affected. Except for Prism, each method was used to predict intensities for both singly- and doubly-charged *b* and *y* ions. (Prism predicts the singly-charged fragment intensities only).

Here, we compare the performance of the evaluated methods in terms of prediction accuracy (separately for tryptic and HLA data) and prediction speed. Note that our comparison is limited to the accuracy and speed of MS/MS fragment intensity prediction. Other functionalities (prediction of retention time, for example) that the included methods might have are not assessed in this study.

### Prediction Accuracy: Tryptic Peptides

The similarity measures (correlations and normalized angles) between the predicted and the corresponding experimental intensities were computed for each PSM in our data. The distribution of the similarity measures was then calculated (see Methods) for each of the 36 datasets. For HCD and CID data, the total number of precursors used for evaluation were respectively 875,544 and 193,430 (a total of 1,068,974). For all 29 HCD datasets and for all six methods, the medians and 10, 25, 75, and 90% quantiles of the distributions, calculated using the singly-charged fragment intensities, are given in Table S1. The medians/quantiles of the distributions computed from intensity profiles for both singly and doubly-charged fragments are tabulated in Table S2 (excluding Prism). The corresponding values for the 7 CID sets are given in Tables S3 (for singly-charged fragments only) and S4 (for both singly and doubly-charged fragments). Note that Tables S3 and S4 also include the medians/quantiles for pDeep3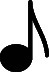 and PeptDeep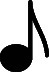 (CID). For HCD data, these values are given in Table S5.

Since some of the methods could not be applied to both HCD and CID data, we decided to make comparisons for these dissociation types separately. Moreover, we found out that the predicting power and ranking of the methods depend not only on the dissociation type but also on the instrument used. Thus, in this subsection, we provide separate comparisons and ranking for 4 different combinations of dissociation type and instruments. Of note, we observed a significant level of variability in the distributions and their medians from set to set, see Tables S1–S5, so combining several datasets from different PRIDE submissions appears to be necessary for getting an objective assessment of prediction accuracy. At the end of this section, we also assess the effect of PTMs (only methionine oxidation), peptide charge, peptide length, and collision energy on the prediction accuracy of the methods.

#### Overall Comparison (HCD)

Since the performance of the methods is instrument-dependent, we realize that the imbalance between the numbers of datasets with different instruments (23 QE, 10 Fusion-Lumos, 3 Velos; see Table 1), while reflecting current instrument usage across the mass spectrometry field, could lead to biased results favoring methods that perform better on QE-acquired datasets. Hence, overall comparisons were made separately for different instruments. Specifically, the 29 HCD nonredundant sets of precursors were partitioned into two groups based on the instrument used for obtaining the data (23 QE and 6 Fusion-Lumos sets). For each of the two groups and each of the six methods, the distributions of the similarity measures between the predicted and experimental spectra were found and combined to generate an overall distribution (see Methods for details).

**Figure 2:**
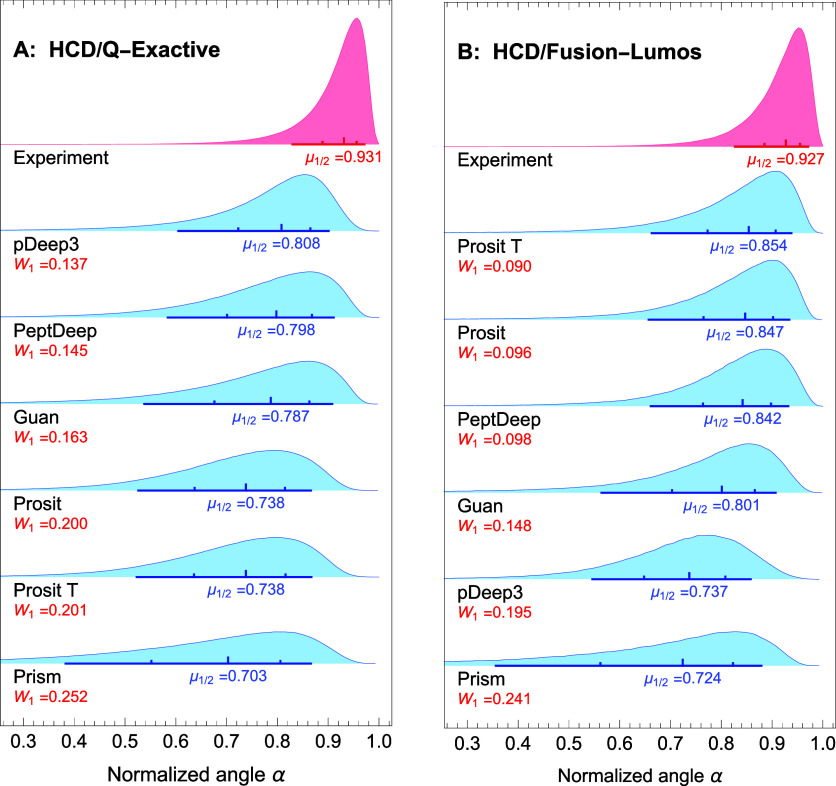
Overall comparison between the methods for HCD dissociation. The distributions of similarity between the predicted and experimental intensity profiles as measured by the normalized angle (the “predicted” distributions) are plotted (in blue) for different methods for the QE (A) and Fusion-Lumos instruments (B). To be able to include Prism in the figure, only singly-charged fragment intensities were used for calculating the normalized angles. In each panel, the distribution of the normalized angles between the reference experimental profiles and their corresponding experimental profiles (the “experimental” distribution; see text) is also plotted (in red) at the top. On each panel, the predicted distributions are ordered, based on their medians (μ_1/2_) and in descending order, from top to bottom. For each distribution, the median is shown by the largest tick on the line. The smaller ticks depict 25 (left) and 75 (right) percentiles. The left and right ends of the line show 10 and 90 percentiles, respectively. The Wasserstein’s (the earth mover’s) distances *W*_1_ between the predicted and experimental distributions are also shown for each of the evaluated methods. Prosit T denotes Prosit Transformer.

The combined distributions of the normalized angles between the predicted and corresponding experimental spectra are shown in blue in Figure 2A,B for QE and Fusion-Lumos, respectively. Since Prism predicts the intensities for singly-charged fragments only, we followed this restriction when generating the distributions. These distributions, which we refer to as the “predicted” distributions, are ranked based on their medians from top to bottom (in descending order) in both figures. For comparison, combined distributions of the normalized angles between the reference experimental spectra and all experimental spectra associated with the same precursor (see the Similarity Measures subsection) are also shown in red at the top of the figures. We henceforth refer to these distributions as the “experimental” distributions. The total number of precursors used to generate the predicted (experimental) distributions were respectively 705,657 (454,457) and 169,887 (116,839) for Figure 2A,B. For a more complete comparison, in addition to the medians (denoted by μ_1/2_), the *W*_1_ Wasserstein’s distances between the predicted and experimental distributions are also shown in both figures. Of note, we also generated these figures using correlation as the similarity measure (Figure S1A,B), which show the same trends as those seen in Figure 2A,B. Therefore, here we discuss only the distributions of the normalized angles.

Comparing the predicted distributions with the experimental ones, Figure 2A,B demonstrate that generally the evaluated methods perform well, although there is still room for improvement, especially for data obtained using QE. This is evident from the rather large distance (*W*_1_ = 0.137 and *W*_1_ = 0.090 in panels A and B, respectively) between the experimental and the top-ranking predicted distributions and also from the significant difference between the corresponding medians (μ_1/2_ = 0.931 vs μ_1/2_ = 0.808 in panel A, and μ_1/2_ = 0.927 vs μ_1/2_ = 0.854 in panel B).

Comparing the predicted distributions with each other, Figure 2A,B show two different trends. For QE data (Figure 2A), pDeep3 is the top-ranking method, although PeptDeep and Guan’s method are close second and third, respectively. With almost identical distributions, Prosit and Prosit Transformer are tied in fourth place above Prism. In Figure 2B, on the other hand, Prosit Transformer and pDeep3 switch places, and Prosit is a very close second. The fact that Prosit performs better for Fusion instruments has also been reported by Xu et al.^11^ The significant difference between the prediction accuracy of Prosit and Prosit Transformer for QE and Fusion-Lumos data is presumably due to the fact that these methods have been trained using only Fusion-Lumos data. On the contrary, the other transformer-based method, i.e., PeptDeep, consistently performs well for the two types of instruments and its prediction accuracy in both cases is close to that of the top-ranking methods, indicating it is a good all-around method.

Of note, Figure 2 indicates that Prosit and Prosit transformer (based on Prosit), have almost identical overall predicted distributions for QE and very close ones for Fusion-Lumos data. This closeness is not limited to the overall distributions, the two methods also result in very close median normalized angles for each of the nonredundant sets of precursors (Table S1; average difference in median is 0.004). In fact, we found that Prosit and Prosit Transformer predict very similar spectra. Computing the normalized angle between the spectra predicted by the two methods for all precursors in our data, we found the median angle to be 0.938° (with 95% of the normalized angles larger than 0.808).

Figure 2A,B indicates that Prism does not perform as well as other models for both the QE and Fusion-Lumos data. However, one should keep in mind that, among the six evaluated methods, Prism is the only one that is trained to be used for predicting the intensities for both HCD and CID data. (Prosit has two separate models for HCD and CID data, and pDeep3/PeptDeep can be fine-tuned to handle CID data but fine-tuning effectively changes the model). This design feature might explain the fact that Prism is not as good as the other models. Indeed, one expects models trained specifically for one type of data to do better than models that have been trained to handle various types.

Another interesting observation from the figures is that all predicted distributions and even the experimental ones have long tails. This is presumably due to misidentified peptides (reported false discovery rate of 1%) or too many missing peaks in the experimental spectra. We did not filter the MaxQuant results based on any spectrum quality score. This may have contributed to the elongation of the tails. However, we expect the noisy spectra to affect all prediction methods in a similar manner, so the presence of such spectra should not change the conclusions of this paper.

#### Effect of Fine Tuning/NCE Calibration (HCD)

**Figure 3:**
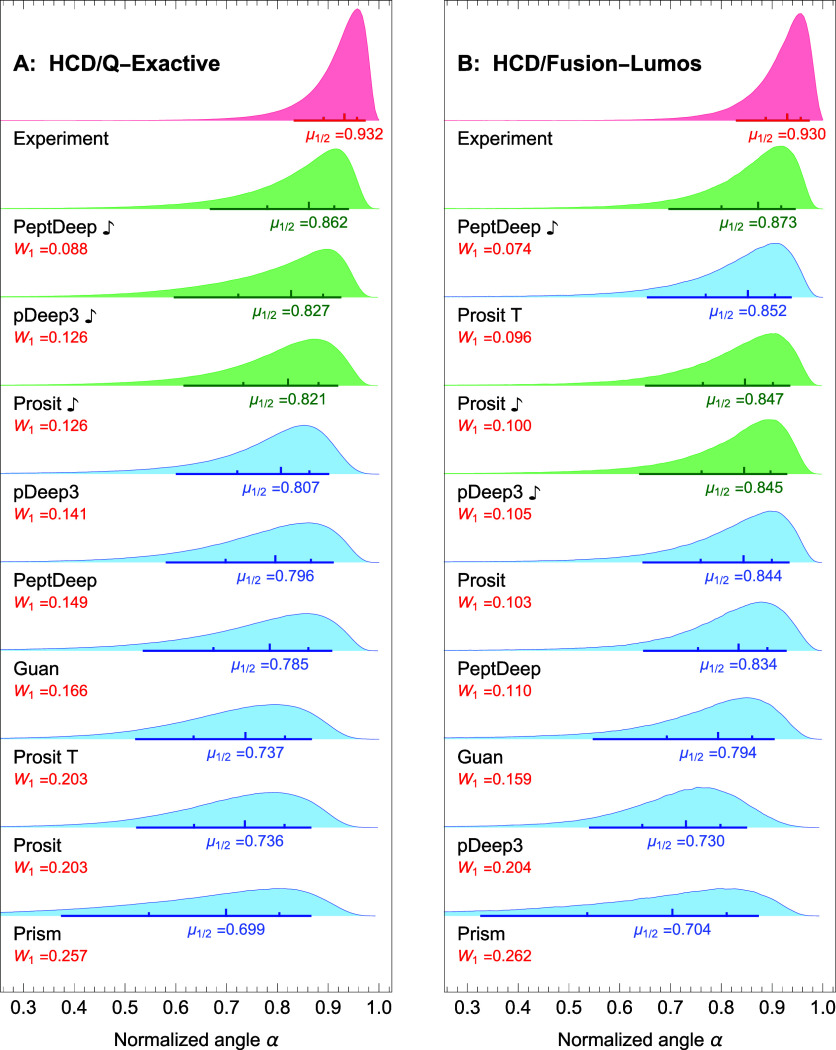
Effect of fine-tuning for HCD dissociation. The similarity distributions for Prosit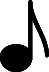, pDeep3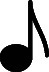, and PeptDeep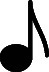 (in green) are compared with those obtained using the other methods for QE (A) and Fusion-Lumos (B) instruments. The notations are the same as in Figure 2.

Although NCE-calibration of Prosit is recommended for HCD data, it requires more time and effort as well as an additional (albeit small) set of PSMs. Because Prosit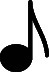 (NCE-calibrated version of Prosit) receives additional information, i.e., the small set of PSMs used for calibration, comparing it with the other methods may not be entirely fair, so we decided to use noncalibrated Prosit when generating Figure 2A,B. Our rationale was to compare the performance of out-of-the-box tools, in the form they are available for download and use by end users. On the other hand, some users may prefer to invest extra time and effort in order to obtain more accurate intensity profiles, so it is important to to see how much improvement is achieved by Prosit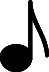 when compared with Prosit. Thus, we used Prosit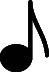 and recomputed the distributions of the normalized angles. Since pDeep3 and PeptDeep also offer fine-tuning as a feature, we fine-tuned these two methods and regenerated the corresponding distributions. These distributions, colored in green, are compared with those obtained using the other methods in Figure 3A,B for QE and Fusion-Lumos, respectively (the distributions are reranked based on their medians). The medians/quantiles for the individual datasets are given in Table S5.

Note that we had to exclude some of the precursors for fine-tuning/NCE-calibration (see Methods), which reduced the total number of precursors to respectively 601,830 and 123,940 for QE and Fusion-Lumos data. (The numbers of precursors used for generating the experimental distributions were also decreased to 361,569 and 77,560 for QE and Fusion-Lumos, respectively.) Due to this exclusion, Figures 2 and 3 are not exactly comparable, but the differences between the medians shown on the two figures for the unchanged methods (Guan, Prosit Transformer, and Prism) are generally inappreciable. Figure 3A,B indicate that, except for Prosit applied to Fusion-Lumos data, fine-tuning/NCE-calibration improves the prediction accuracy noticeably. Specifically, PeptDeep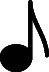 is the top-ranking method for both types of instruments. On the other hand, for Fusion-Lumos data, pDeep3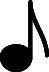 performs significantly (more than 15% larger median) better than pDeep3, but is still in fourth place, although its performance is comparable to those of Prosit Transformer, Prosit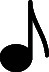, and Prosit. For QE data, pDeep3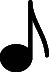 and Prosit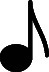 both outperform their out-of-the-box versions but not by enough margin to outperform PeptDeep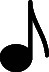.

Interestingly, Figure 3B indicates little difference between Prosit and Prosit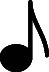 distributions. To explain this observation, we note that NCE-calibration provides an optimal NCE that must be used for running Prosit. We found that, on average, for the 22 QE sets the optimal NCE was 6.36 units larger than the actual NCE, whereas for the 5 Fusion-Lumos sets, the average increase in NCE was 1.2 (in fact, no change was observed for the Fusion sets). This is presumably because Prosit was trained on the Fusion-Lumos data. In other words, Prosit is already optimized for use with Fusion-Lumos data, so NCE-calibration has little effect on the results in this case.

The fact that fine-tuning/NCE-calibration improves the results demonstrates the usefulness of these procedures. However, users should keep in mind that fine-tuning/NCE-calibration demands extra time and effort and requires setting aside a (small) set of PSMs. Also, it is important to note that the comparisons made in Figure 3A,B may not be entirely fair as PeptDeep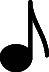, pDeep3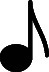, and Prosit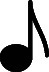 employ extra information (the PSMs used for fine-tuning/NCE-calibration) not seen by other methods. It is likely that any method can be improved by fine-tuning. However, since this option is not available for the other methods, it is not possible to test this hypothesis. On the other hand, the fact that PeptDeep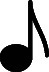, pDeep3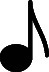, and Prosit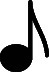 do offer this feature makes them advantageous.

#### Overall Accuracy (CID)

**Figure 4:**
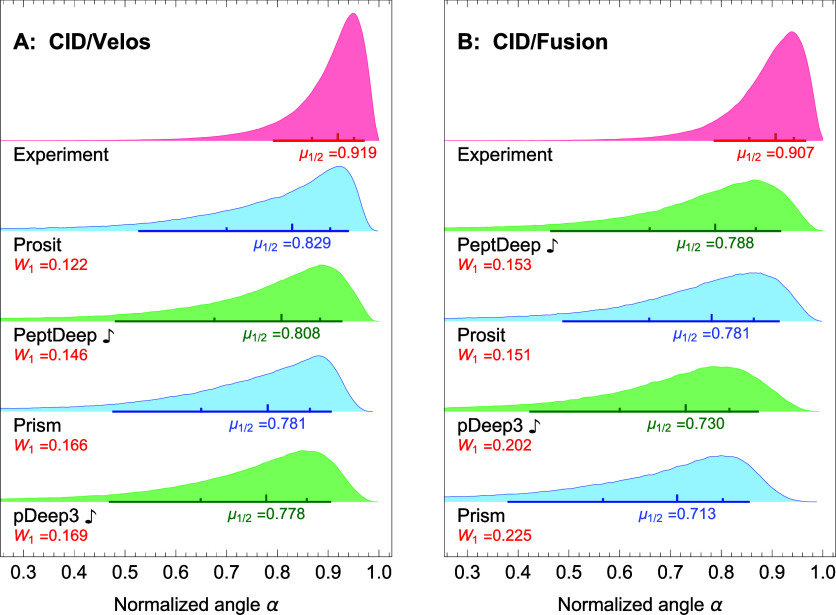
Overall comparison between the methods for CID dissociation. The predicted and experimental distributions of the normalized angles for CID data are plotted in blue/green and red, respectively. The distributions in green are obtained using the fine-tuned versions (marked by 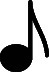) of the methods, whereas the ones in blue are obtained for the out-of-the-box methods.

As mentioned previously, comparisons between fine-tuned methods and other approaches may not be entirely fair. However, since only two out-of-the-box methods (Prosit and Prism) are trained for CID data, we decided to include PeptDeep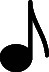 and pDeep3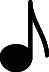 in our overall comparison for CID data. The seven nonredundant sets of CID precursors, containing a total of 193,430 (104,127 Velos and 89,303 Fusion) precursors, were used to evaluate the methods when applied to CID data. Prosit NCE-calibration is recommended only for HCD data, so it was not performed in this case. The ranked computed distributions of normalized angles (calculated using only singly-charged fragment intensities) for pDeep3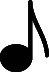, PeptDeep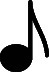, Prosit, and Prism are shown in Figure 4A,B for data obtained using Velos and Fusion instruments, respectively (the other methods have not been trained for use with CID data).

Figure 4A,B indicates smaller medians for the distributions of the normalized angles for PeptDeep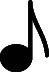, pDeep3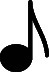, and Prosit compared with the HCD case. On the other hand, Prism performs better on CID/Velos and comparably on CID/Fusion in comparison with its performance on the HCD data (Figure 3), but still ranks below Prosit and PeptDeep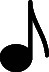 for CID/Velos and below the other three methods for CID/Fusion data. Prosit and PeptDeep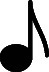 are in the first and second positions for CID/Velos and switch places for CID/Fusion. The two methods, however, have similar predicted distributions with close medians and close Wasserstein’s distances from the experimental distributions in the case of CID/Fusion. We should emphasize again that although PeptDeep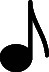 has the advantage of being fine-tunable, it takes extra time and effort to run and requires a portion of PSMs for fine-tuning. Of note, as mentioned previously, the out-of-the-box versions of pDeep3, PeptDeep, Prosit Transformer, and Guan’s method are not included in Figure 4 because they are not trained for CID data. However, for completeness, the performance of these methods on CID data is compared with those of PeptDeep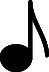, pDeep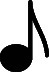, Prosit, and Prism in Figure S2, which indicates poor performance by Prosit Transformer and Guan’s method as well as out-of-the-box versions of pDeep3 and PeptDeep.

It is worth noting that, as in the HCD case, the observed trends in Figure 4 do not depend on the choice of the similarity measure. In other words, for CID data, correlation distributions (Figure S3) indicate the same rankings for the methods as the ones seen in Figure 4, although the median correlations in all cases are higher than median normalized angles. The fact that normalized angles are generally smaller than correlations is expected as the former has been shown to be more sensitive to small dissimilarities between overall similar spectra.^12^ Since the two similarity measures uncover the same trends and given that normalized angle has higher sensitivity, in the rest of the paper we only plot and discuss normalized angles.

The distributions shown in the figures so far have all been calculated by using only singly-charged fragment intensities. The corresponding distributions obtained using both singly and doubly-charged fragment intensities are plotted in Figures S4 and S5 for HCD and CID data, respectively (excluding Prism). Figures S4 and S5 demonstrate the same trends observed in Figures 2 and 4, respectively. However, the median normalized angles are slightly smaller when both singly and doubly-charged fragment intensities are used for calculation. This appears to suggest that (for all methods) it is more difficult to predict the doubly-charged fragment intensities. However, given the difference between the numbers of elements in the intensity profiles (singly-charged only vs singly- and doubly-charged), the normalized angles in the two cases are not exactly comparable. Also, the quality of the spectra when doubly-charged fragments are included may be worse. Since the rankings of the methods were the same regardless of the fragment intensities used for calculating the normalized angles, in the rest of the paper, only normalized angles obtained from singly-charged fragment intensities will be discussed.

#### Effect of Methionine Oxidation

Oxidation of methionine is the only variable PTM considered in this study as it is the only one accepted by most of the assessed methods. (PeptDeep and pDeep3 are the only methods processing other PTMs). To evaluate the effect of this PTM on the prediction accuracy, for the four aforementioned DT/INS combinations, we replotted the distributions of the normalized angles considering only peptides with oxidized methionine (54,470, 12,210, 22,752, 4369 nonredundant precursors with PTM for HCD/QE, HCD/Fusion-Lumos, CID/Velos, and CID/Fusion, respectively). Comparing these distributions (Figures S6 and S7) with those shown in Figures 3 and 4 indicates that all methods perform worse when applied only to precursors with oxidized methionine. However, the drop in the median normalized angle is small (less than 5% change) in most cases and is comparable for most predictors. Thus, the relative rankings of the predictors remain largely the same as before. A clear exception is the Guan’s method that, with the decrease in medians close to 20% for both HCD/QE and HCD/Fusion-Lumos data, ranks last in Figure S6A,B. For HCD data, pDeep3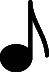 also suffers more than the other methods (although not as much as Guan’s method) and drops in ranking. Interestingly, pDeep3 and pDeep3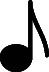 perform comparably for modified peptides in HCD/QE datasets (Figure S6A). This may suggest that pDeep3 should be fine-tuned for modified peptides. However, the developers of pDeep3 concluded that a random mixture of peptides with and without PTMs performs better in fine-tuning, and therefore, we did not fine-tune pDeep3 (or any other method) specifically for modified peptides.

In the case of CID/Velos data, Figure S7A shows the same ranking for the assessed methods as those seen in Figure 4A. On the other hand, Prosit and PeptDeep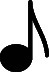 switch places in Figure S7B in comparison with Figure 4B, but they remain the top-2 methods with close median normalized angles in both figures. It is worth noting that the number of modified peptides in our CID/Fusion datasets was rather low (4369), so the rankings may not be as reliable as those shown in Figure 4B.

#### Effect of Precursor Charge *Z*

**Figure 5:**
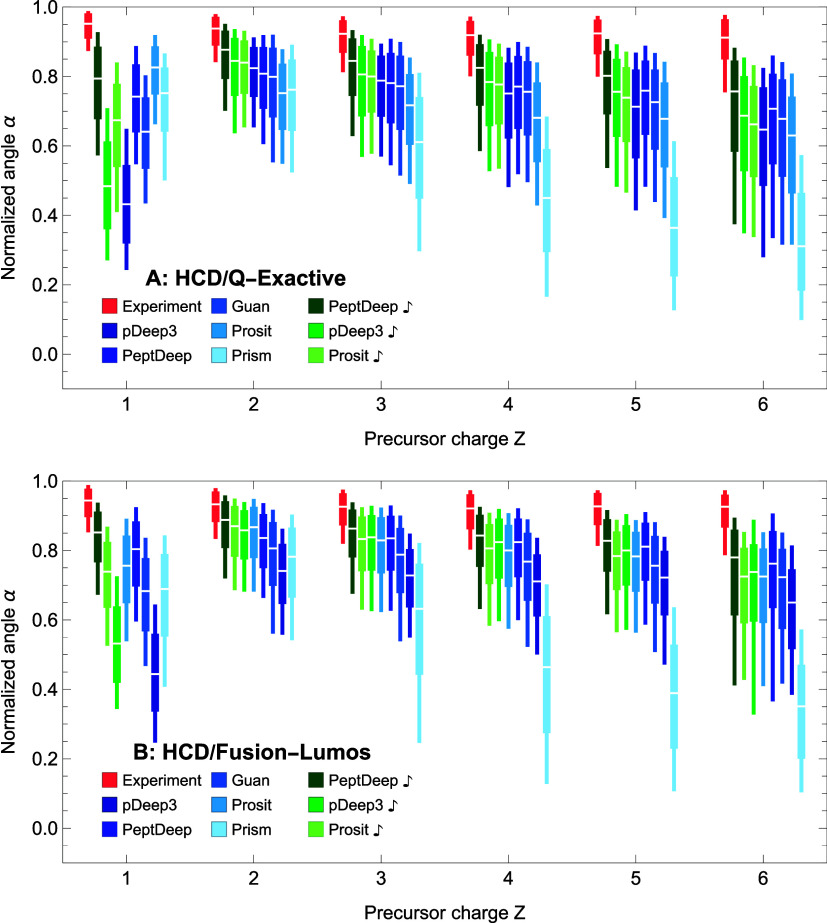
Effect of precursor charge *Z* on similarity distributions for HCD dissociation. For different methods and different charges, quantiles of distributions are shown as box plots for HCD/QE (A) and HCD/Fusion-Lumos (B) data. The plots for out-of-the-box and fine-tuned/calibrated methods are, respectively, in different shades of blue and green. The white line in each box represents the median; the lower/upper end of the box shows the 25/75 percentile, and the lower/upper end of the line shows the 10/90 percentile.

**Figure 6:**
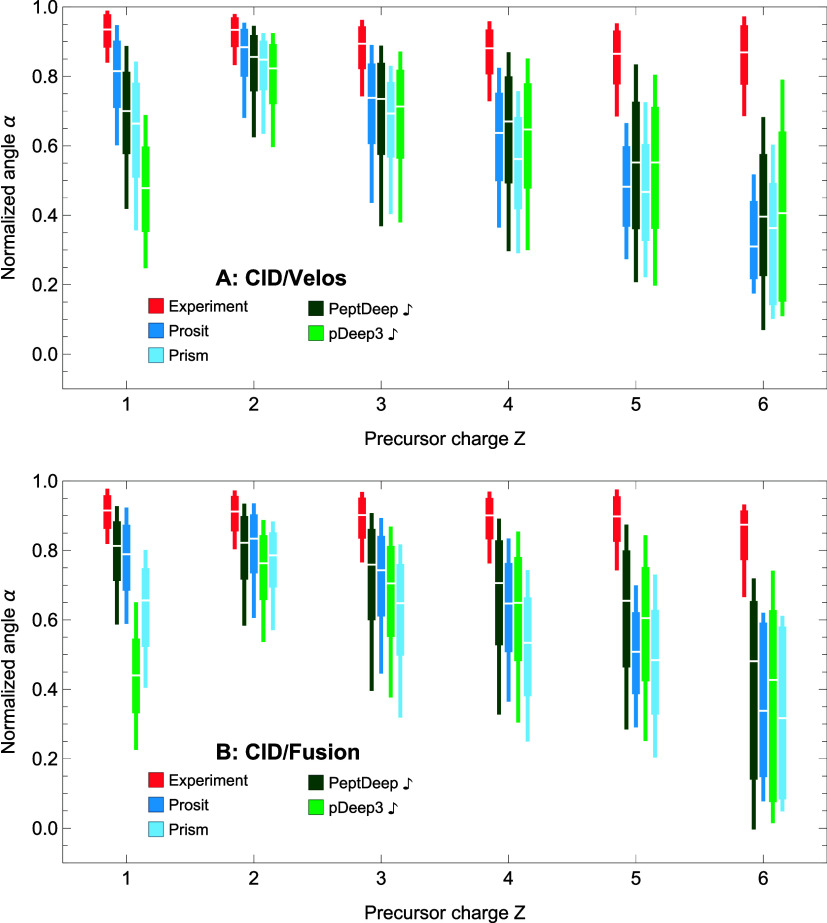
Effect of precursor charge *Z* on similarity distributions for CID dissociation. For different methods and different charges, quantiles of distributions are shown as box plots for CID/Velos (A) and CID/Fusion (B) data. The notations are same as in Figure 5.

To demonstrate the effect of precursor charge on prediction accuracy, quantiles of normalized angle distributions are shown as box plots for charges 1 through 6 in Figures 5A,B and 6A,B for HCD/QE, HCD/Fusion-Lumos, CID/Velos, and CID/Fusion, respectively. As mentioned previously, Prosit Transformer and Prosit produce very similar spectra. Thus, to make Figure 5 clearer, Prosit Transformer is omitted from this figure (and also from the figures in the following subsections discussing the effect of *N* and NCE on prediction accuracy). We chose Prosit (over Prosit Transformer) to be included in the figure because Prosit can be used with both CID and HCD data. The box plots in each panel of Figures 5 and 6 are ordered, from left to right, according to the ranking of their corresponding distributions in the corresponding panel of Figures 3 and 4, respectively. (For example, the left to right order in Figure 5A is the same as the top to bottom order in Figure 3A.) Figures 5A,B and 6A,B demonstrate that in general the per-charge ranking of the methods differs from their overall ranking, although in some cases, the overall ranking is also reflected here. For example, PeptDeep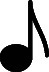 consistently ranks higher than the other methods across all charges for HCD data.

A general pattern seen in Figures 5 and 6 is that the methods perform best for *Z* = 2 and that the medians drop for other charges (especially in the case of CID data; Figure 6). This drop increases for higher (than 2) precursor charges and is also large for *Z* = 1. Interestingly, for *Z* = 1 and HCD data, Figure 5A,B indicates that Prosit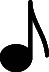 performs worse than Prosit. The fact that the methods perform best for peptides with *Z* = 2 is not surprising as these peptides are the most frequent, so the models can be trained better for these peptides.

#### Effect of Peptide Length *N*

**Figure 7:**
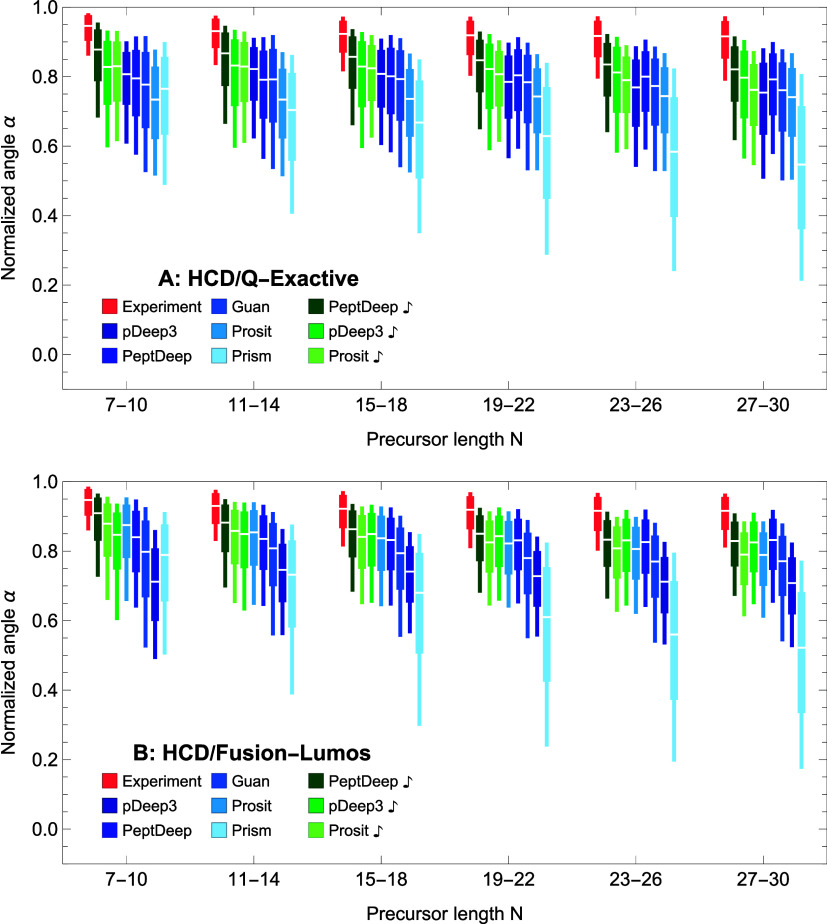
Effect of peptide length *N* on similarity distributions for HCD dissociation. For different methods and different length intervals, quantiles of distributions are shown as box plots for HCD/QE (A) and HCD/Fusion-Lumos (B) data. The notations are the same as in Figure 5.

**Figure 8:**
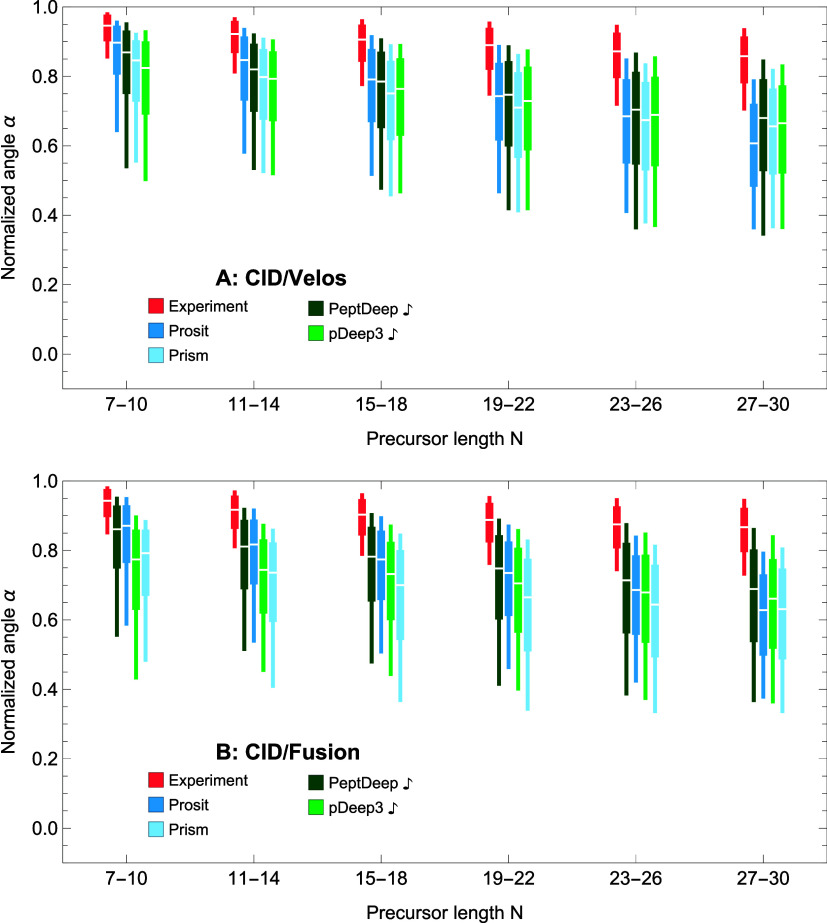
Effect of peptide length *N* on similarity distributions for CID dissociation. For different methods and different length intervals, quantiles of distributions are shown as box plots for CID/Velos (A) and CID/Fusion (B) data. The notations are same as in Figure 5.

Similar to the dependence on precursor charge, we plot quantiles of normalized angle distributions as box plots for 6 different length intervals in Figures 7A,B and 8A,B for HCD/QE, HCD/Fusion-Lumos, CID/Velos, and CID/Fusion, respectively. The methods are ordered, from the left to right, in the same way, as described in the previous subsection. As expected, the figures show that the prediction accuracy of all methods suffers as the peptide length increases, although the drop in medians is not as pronounced as the ones observed for *Z* > 2. Interestingly, for the HCD data, PeptDeep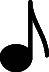 outperforms other methods across all length intervals. In the CID case, however, Prosit beats PeptDeep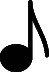 for shorter (*N* ≤ 14) precursors whereas for longer (*N* ≥ 19) sequences PeptDeep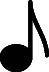 performs better. For precursors of length 15–18, the two methods (PeptDeep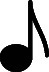 and Prosit) have close medians with PeptDeep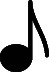 (Prosit) being slightly better for CID/Fusion (CID/Velos) data.

#### Effect of NCE

**Figure 9:**
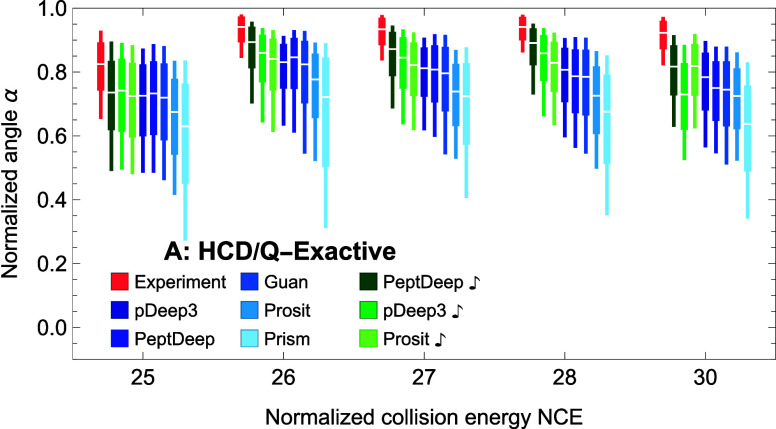
Effect of normalized collision energy on similarity distributions for HCD dissociation with a QE mass spectrometer. For different methods, quantiles of distributions are shown as box plots. The notations are same as in Figure 5.

We investigated the dependence of prediction accuracy on NCE for HCD/QE data only because this was the only INS/DT combination for which we had several NCE values and more than one set of precursors per each NCE value (Table 1). The quantiles of normalized angle distributions are plotted as box plots as functions of NCE in Figure 9 for different methods (in the same order as in Figure 3A). The figure suggests that, regardless of the prediction method used, the dependence of prediction accuracy on NCE is not as strong as its dependence on precursor charge or length, especially for NCE ≥ 26. For NCE = 25 the normalized angles are significantly lower. However, in this case, the experimental box plot (in red) is also significantly lower, which may be a problem specific to the two HCD/QE sets with NCE = 25 we have used.

### Prediction Accuracy: HLA Peptides

The methods were also compared based on their performances in predicting the fragment intensity profiles of HLA (nontryptic) peptides. We used the aforementioned 14 HLA datasets (11 HCD and 3 CID; see Methods and Table 2) to assess each method in the same way as we did for tryptic data. Since we have already investigated the effect of fine-tuning and NCE-calibration, here we evaluate the performances of all 9 methods (the six methods + PeptDeep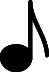, pDeep3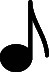, and Prosit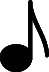) for HCD data, and those of Prosit, Prism, PeptDeep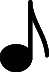, and pDeep3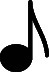 for CID data. After excluding the precursors used for fine-tuning/NCE-calibration (and also peptides with charges larger than 6 or lengths longer than 30) we were left with 466,769, 108,449, and 37,147 (total of 612,365) nonredundant precursors for evaluation of the methods on HCD/QE, HCD/Lumos, and CID/Lumos, respectively.

**Figure 10:**
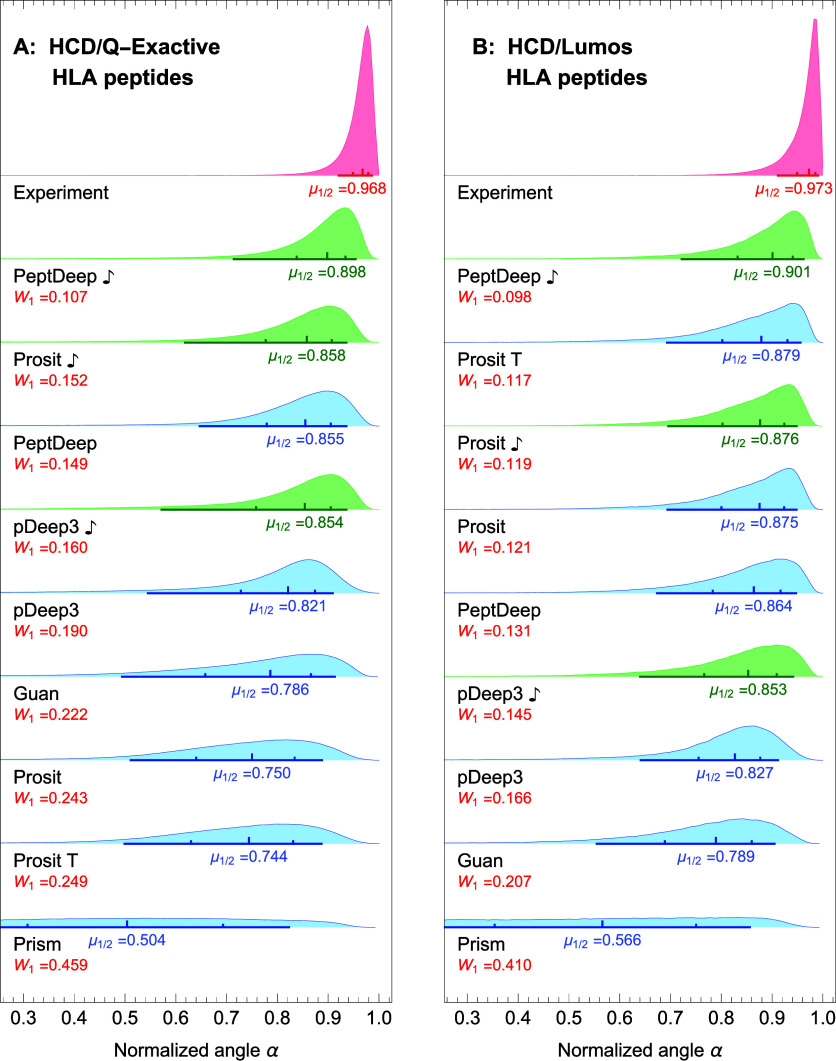
Comparison between performance of the methods for HLA peptides for HCD dissociation. The distributions of normalized angles are plotted for each method for the HCD/QE (A) and HCD/Lumos (B) HLA data. The notations are same as in Figure 2.

**Figure 11:**
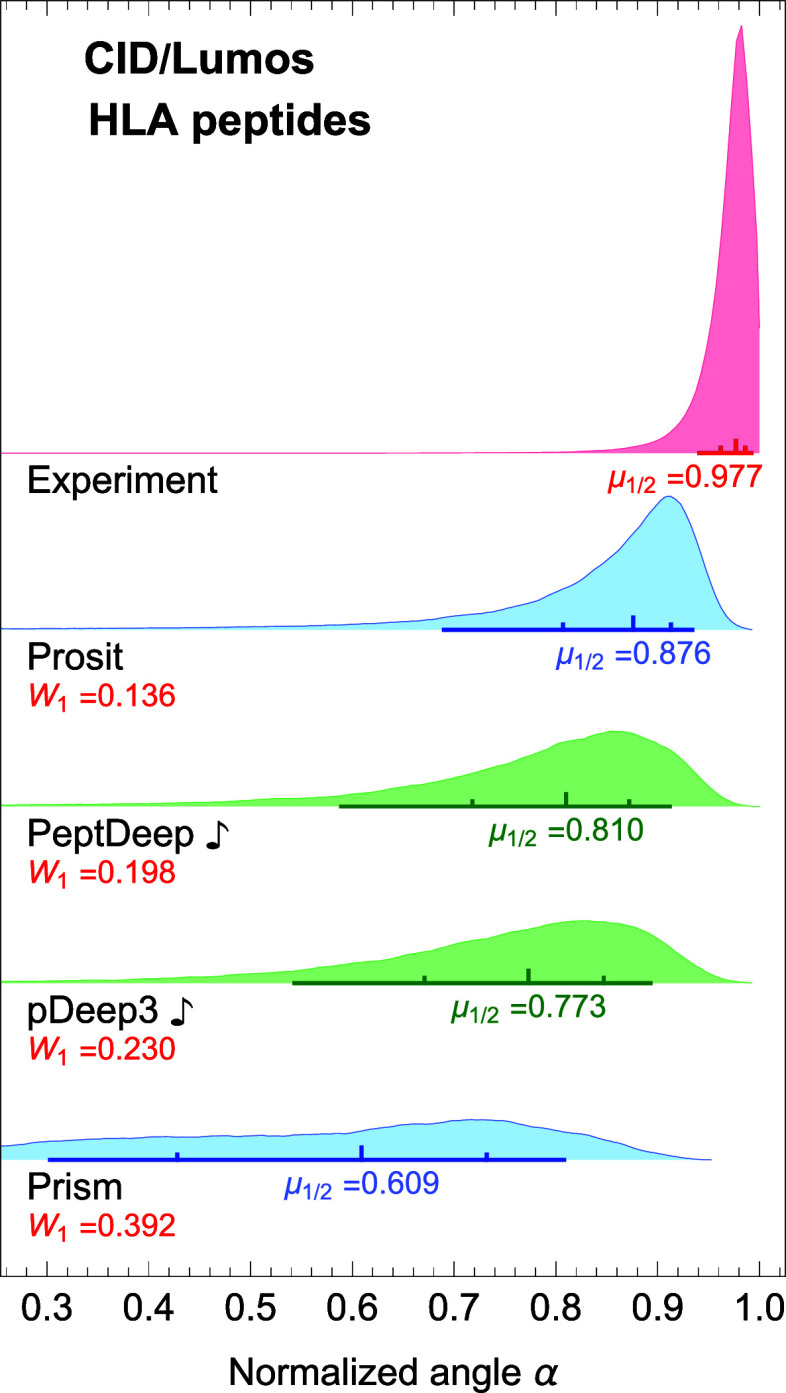
Comparison between the performance of the methods for HLA peptides for CID dissociation. The distributions of normalized angles are plotted for each method for CID/Lumos HLA data. The notations are same as in Figure 2.

The distributions of normalized angles are shown in Figures 10A,B, and 11 for HCD/QE, HCD/Lumos, and CID/Lumos, respectively. Comparing Figure 10A,B with the corresponding figures showing the distributions for tryptic peptides (Figure 3A,B) indicates that, for HCD data, in most cases, the relative rankings of the methods are the same for tryptic and HLA data. Specifically, for both HCD/Fusion-Lumos and HCD/QE, PeptDeep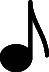 is still the top-ranking method, and in the case of HCD/Fusion-Lumos the top three methods remain exactly the same. For HCD/QE on HLA data, pDeep3 and pDeep3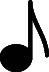 have both moved down below Peptdeep making Prosit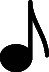 and PeptDeep respectively number 2 and 3 methods, although Prosit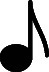, PeptDeep, and pDeep3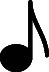 have very close medians in this case. For CID data, in comparison with the tryptic case (Figure 4B), Prosit and PeptDeep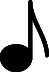 switch places with the former in first place and the latter in second place. (Note that Lumos in this paper refers to the Fusion Lumos instrument, that belongs to the Fusion family of mass spectrometers.)

An interesting observation from Figures 10A,B and 11 is that the reported medians are mostly larger than the corresponding ones shown in Figures 3A,B and 4B. However, this does not mean that most methods perform better on HLA data. Indeed, a comparison of the *W*_1_ distances between the predicted and experimental distributions indicates that they are larger for HLA data. This suggests that the methods actually perform slightly worse than in the tryptic case.

### Prediction Speed

**Figure 12:**
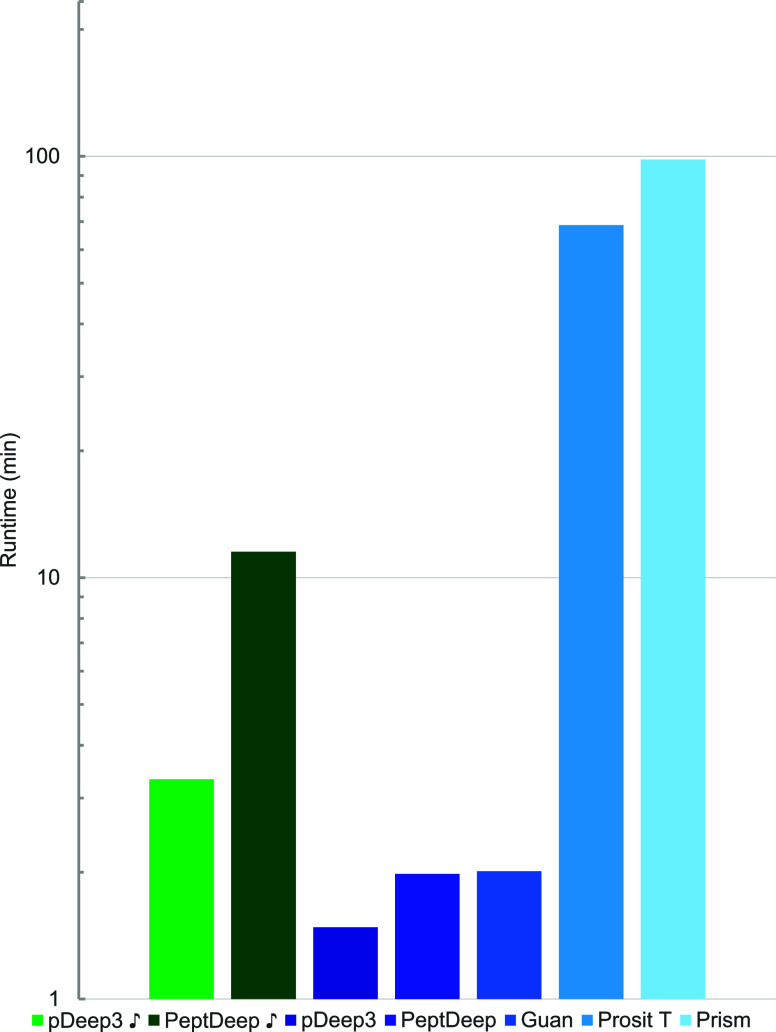
Comparison of run times. The average run times for the methods are plotted. As before, the fine-tuned versions are plotted in green, and the rest are plotted in blue. Note that the scale on the vertical axis is logarithmic.

Although deep learning-based methods run significantly faster on GPUs (graphics processing units), we used CPUs (central processing units) for benchmarking the prediction speed of the assessed methods. The rationale behind this decision is that GPUs may not be readily available to everyone, so we expect the majority of users to utilize CPUs for running these methods. Each method was run using eight CPUs on the Biowulf cluster of the National Institutes of Health. (Since the prediction speed is not expected to differ for tryptic and HLA data, speed evaluation was performed using only the tryptic datasets). To have a realistic comparison between run times, we recorded the time spent on reading and processing the input csv files, predicting, and writing the results. The measured run times are given Table S6 for all nonredundant sets of precursors. The averaged (over all sets of precursors) run times are plotted in Figure 12. These average values give the users an idea of how long a “typical” run would take. Figure 12 indicates pDeep3 as the fastest method followed closely by PeptDeep and Guan’s method. On the other hand, the figure shows that it takes much longer to run Prosit Transformer and Prism. The slower speed of Prosit Transformer is also reported by its developers.^17^ It is worth noting that Prism needs to be run on the Google Cloud in either the “online” or “batch” mode. The online mode is easier to set up and run, but one can submit only 100 precursors at a time for prediction. The batch mode does not have this limitation but is more difficult to set up and requires additional information from the users that may raise privacy concerns. We chose the online method and submitted the data in sets of 100 precursors, which perhaps resulted in a long run time.

Of note, since Prosit was run on its own web server, it was not possible to measure the run times for Prosit and Prosit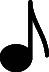. (Prosit does not report the running time, and one has to keep refreshing the web page until the results are ready). For this reason, Prosit and Prosit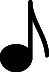 are missing from Figure 12. However, although the measurement of run time was not possible, we can report that Prosit typically took a few minutes to report the results.

Also shown in Figure 12 are the average run times for pDeep3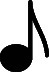 and PeptDeep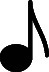. As expected, the figure shows that one needs to spend a significant amount of time fine-tuning pDeep3 and PeptDeep, with an especially large increase in run time for PeptDeep. Therefore, one should take the extra time needed into account when deciding whether to fine-tune these methods.

## Conclusions

In this study, six deep learning-based methods for predicting *b* and *y* MS/MS fragments intensity profiles are comprehensively evaluated and compared with each other. We used fragmentation profiles of tryptic peptides with or without PTMs (called below tryptic-all), tryptic peptides with PTMs (tryptic-modified), and HLA nontryptic peptides for our evaluations.

Our results suggest that none of the methods can be regarded as best-performing under all circumstances. PeptDeep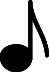 outperforms all other methods for HCD data in all cases. Prosit performs better for CID data in all cases, except for CID/Fusion-Lumos/tryptic-all (Figure 4B). Note that Prosit Transformer is a tiny bit better than Prosit for HCD/Fusion-Lumos data, but generally predicts spectra that are very similar to those predicted by Prosit. Also, in the case of CID/Fusion-Lumos/tryptic-all, Prosit loses to PeptDeep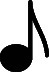, but it is a close second (median normalized angles of 0.788 and 0.781 for PeptDeep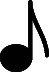 and Prosit, respectively), so one can consider Prosit to be the overall best-performing method for CID data. We thus recommend using PeptDeep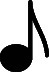 for HCD and Prosit for CID data, although one might benefit slightly from employing PeptDeep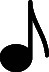 for CID/Fusion-Lumos/tryptic data. Of note, for CID/Fusion-Lumos/tryptic-modified we had fewer than 5000 precursors to test the methods with, so the results in this case may not be as reliable as in others.

Although fine-tuning PeptDeep and pDeep3 requires more time/effort and additional data, it does improve the results and so is recommended. We found that the NCE-calibration of Prosit (applicable to only HCD data) has almost no effect for HCD/Fusion-Lumos data. For HCD/QE data, NCE-calibration improves the performance of Prosit but not by enough margin to outperform PeptDeep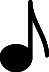, so, as mentioned previously, we recommend PeptDeep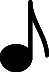 for HCD/QE data. However, if an investigator decides to use Prosit for HCD/QE data, then we do recommend NCE-calibration.

Although we recommend fine-tuning, in some cases, an investigator may not want to go through the fine-tuning process, so it is worth comparing the out-of-the-box performance of the methods. Our results suggest that (out-of-the-box) Prosit outperforms all other methods except for the case of HCD/QE data, so it is recommended for usage, except in this case. On the other hand, although PeptDeep performs well for HCD data, ranks high among the out-of-the-box methods, and is close (in terms of median normalized angle) to the top-ranking methods, it only ranks first for HCD/QE/HLA data. For tryptic peptides, it is pDeep3 that takes the first place, according to our results. In the absence of fine-tuning, we therefore recommend using pDeep3 (PeptDeep) for tryptic (HLA) peptides and PeptDeep (pDeep3) as a close alternative.

As for prediction speed, our results suggest that the top-ranking out-of-the-box methods (Prosit, PeptDeep, and pDeep3) are comparable. Although we do not have an exact assessment of prediction speed for Prosit, we note that all of these methods finish a typical run within a couple of minutes. PeptDeep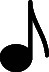 is significantly slower than its corresponding out-of-the-box version, but it still runs in a reasonable time frame, and improves the result significantly. Thus, we believe the extra time and effort required for fine-tuning is worth it.

## Supporting Information Available

Similarity distributions for HCD dissociation, as measured by Pearson’s correlations; similarity distributions for CID dissociation, for all methods; similarity distributions for CID dissociation, as measured by Pearson’s correlations; similarity distributions for HCD dissociation, for both singly and doubly-charged *b* and *y* fragments; similarity distributions for CID dissociation, for both singly and doubly-charged *b* and *y* fragments; Similarity distributions for HCD dissociation, only modified peptides; and similarity distributions for CID dissociation, only modified peptides (PDF)
Quantiles (10, 25, 50, 75, and 90%) for all HCD sets, singly-charged *b* and *y* fragments only; quantiles (10, 25, 50, 75, 90%) for all HCD sets, singly- and doubly-charged *b* and *y* fragments; quantiles (10, 25, 50, 75, 90%) for all CID sets, singly-charged *b* and *y* fragments only; quantiles (10, 25, 50, 75, and 90%) for all CID sets, singly and doubly-charged *b* and *y* fragments; quantiles (10, 25, 50, 75, 90%) for all HCD sets, including fine-tuned/calibrated methods, singly-charged *b* and *y* fragments only; and run times for all methods/datasets (XLSX)
