# Supplementary material for: Systematic Assessment of Deep Learning-Based Predictors of Fragmentation Intensity Profiles
Source: J Proteome Res. 2024 May 10;23(6):1983–99. doi: 10.1021/acs.jproteome.3c00857 (PMC11165591; doi:10.1021/acs.jproteome.3c00857)
Supplement: Supplementary file 1 — pr3c00857_si_001.pdf [file pr3c00857_si_001.pdf]

Supplemental figures for "Systematic assessment of deep learning-based predictors of fragmentation intensity profiles"

Mehdi B Hamaneh[1], Aleksey Y. Ogurtsov[1], Oleg I. Obolensky[1], and Yi-Kuo Yu[1]

[1]National Center for Biotechnology Information, National Library of Medicine, National Institutes of Health, Bethesda, MD 20894, USA

Table of contents

- Figure S1: Similarity distributions for HCD dissociation, as measured by Pearson's correlations;
- Figure S2: Similarity distributions for CID dissociation, for all methods;
- Figure S3: Similarity distributions for CID dissociation, as measured by Pearson's correlations;
- Figure S4: Similarity distributions for HCD dissociation, for both singly- and doubly-charged b and y fragments;
- Figure S5: Similarity distributions for CID dissociation, for both singly- and doubly-charged b and y fragments;
- Figure S6: Similarity distributions for HCD dissociation, only modified peptides;
- Figure S7: Similarity distributions for CID dissociation, only modified peptides;
- Table S1 (XLSX): quantiles (10%, 25%, 50%, 75%, 90%) for all HCD sets, singly-charged b and y fragments only;
- Table S2 (XLSX): quantiles (10%, 25%, 50%, 75%, 90%) for all HCD sets, singly- and doubly-charged b and y fragments;
- Table S3 (XLSX): quantiles (10%, 25%, 50%, 75%, 90%) for all CID sets, singly-charged b and y fragments only;
- Table S4 (XLSX): quantiles (10%, 25%, 50%, 75%, 90%) for all CID sets, singly- and doubly-charged b and y fragments;
- Table S5 (XLSX): quantiles (10%, 25%, 50%, 75%, 90%) for all HCD sets, including fine-tuned/calibrated methods, singly-charged b and y fragments only;
- Table S6 (XLSX): run times for all methods/datasets.

Figure S1

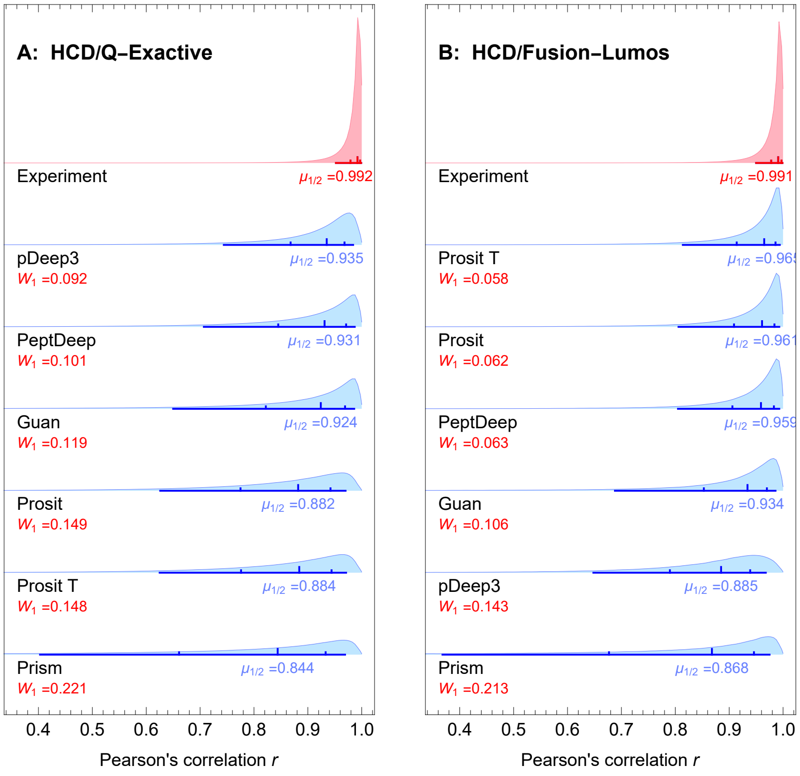

Figure S1: Overall comparison between the methods (HCD; correlations; singly-charged). This is a revised version of Figure 2 of the main text with the distributions of correlation (between the predicted and experimental spectra) replacing the distributions of normalized angle. The distributions for HCD/QE and HCD/Fusion-Lumos are respectively shown in panels A and B. The figure shows the same trends observed in Figure 2 of the main text.

FIgure S2

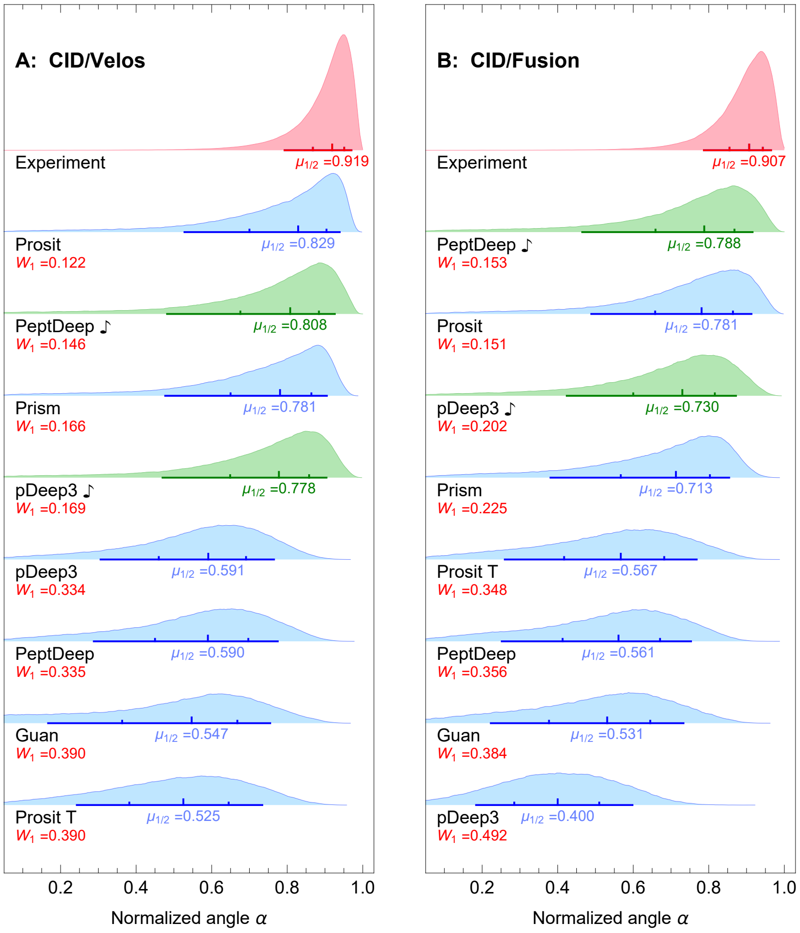

Figure S2: Overall comparison between the methods (CID). For comparison the distributions of normalized angle for pDeep3, PeptDeep (the out-of-the-box versions), Prosit Transformer, and Guan's method are added to what is shown in Figure 4 of the main text. Panels A and B respectively show the distributions for CID/Velos and CID/Fusion data. As expected, these methods, which are not trained for CID data, perform poorly on this type of data.

Figure S3

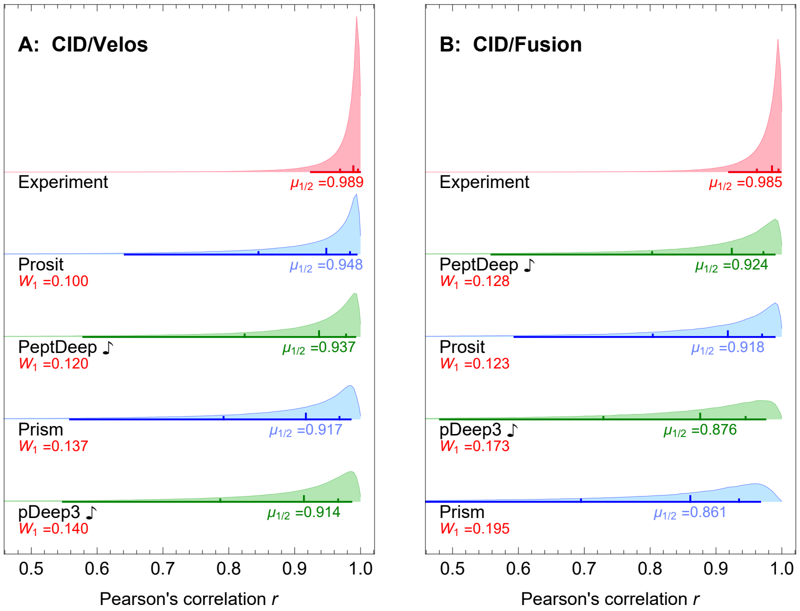

Figure S3: Overall comparison between the methods (CID; correlations; singly-charged). This is a revised version of Figure 4 of the main text with the distributions of correlation (between the predicted and experimental spectra) replacing the distributions of normalized angle. The distributions for CID/Velos and CID/Fusion are respectively shown in panels A and B. The figure shows the same trends observed in Figure 4 of the main text.

Figure S4

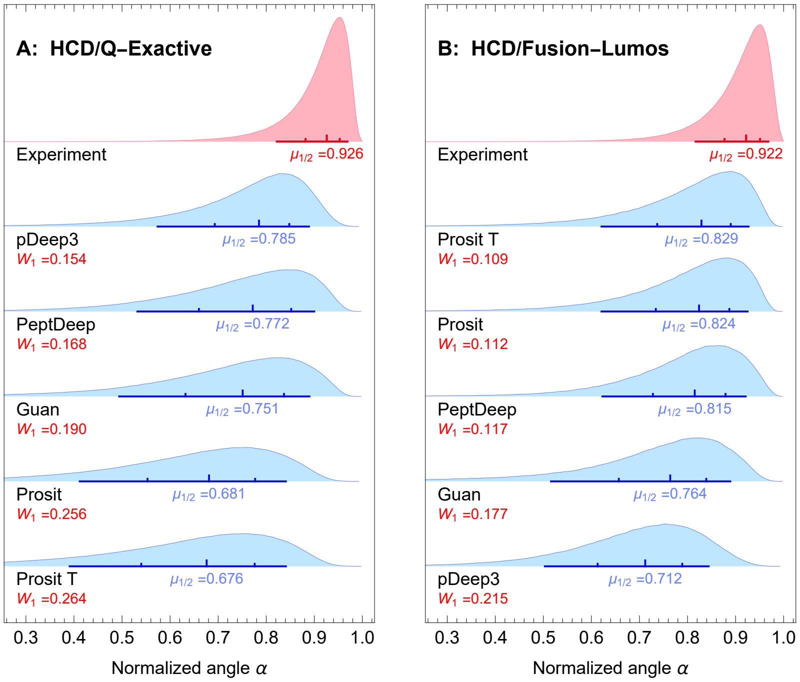

Figure S4: Overall comparison between the methods (HCD; singly- and doubly-charged fragments). The figure shows the distributions plotted in Figure 2 of the main text, but both singly- and doubly-charged fragment intensities have been used to calculate the normalized angles between the predicted and experimental spectra. The distributions for HCD/QE and HCD/Fusion-Lumos are respectively shown in panels A and B. Since Prism does not predict the intensities for doubly-charged fragments, it has been omitted from this figure. The figure shows the same trends as observed in Figure 2 of the main text.

Figure S5

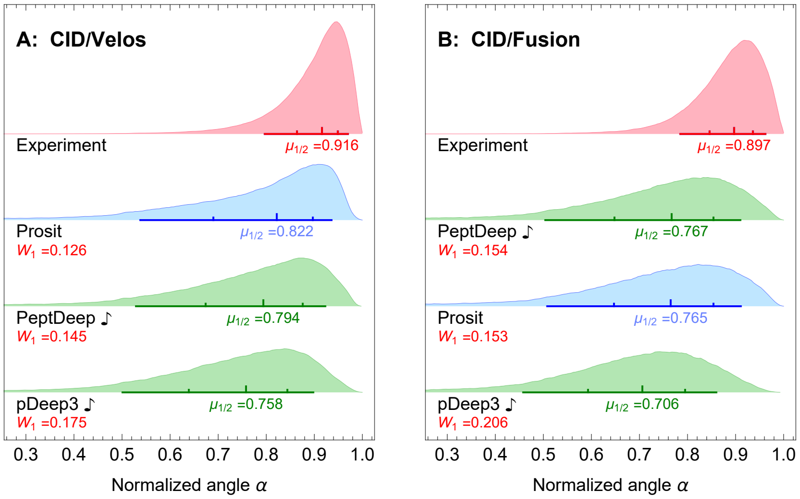

Figure S5: Overall comparison between the methods (CID; singly- and doubly-charged). The figure shows the distributions plotted in Figure 4 of the main text, but both singly- and doubly-charge fragment intensities have been used to calculate the normalized angles between the predicted and experimental spectra. The distributions for CID/Velos and CID/Fusion are respectively shown in panels A and B. Since Prism does not predict the intensities for doubly-charged fragments, it has been omitted from this figure. The figure shows the same trends observed as in Figure 4 of the main text.

Figure S6

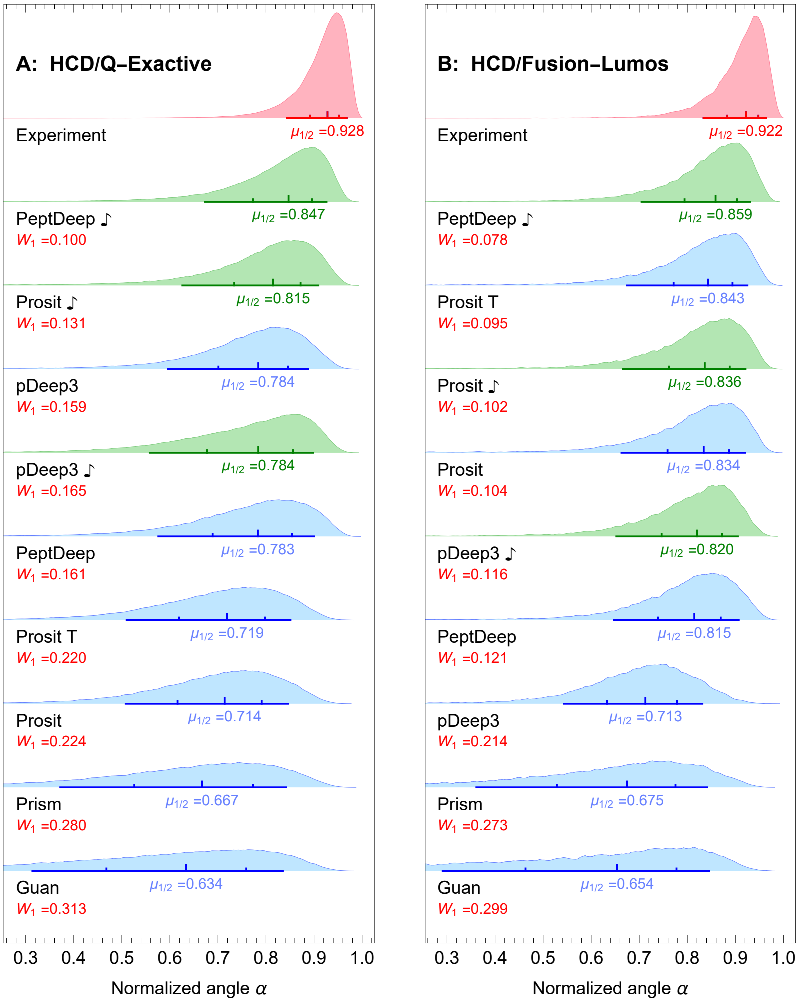

Figure S6: The effect of methionine oxidation (HCD; singly-charged). The figure shows the distributions of normalized angle for different methods when applied to peptides with methionine oxidation for HCD/QE (A) and HCD/Fusion-Lumos data (B).

Figure S7

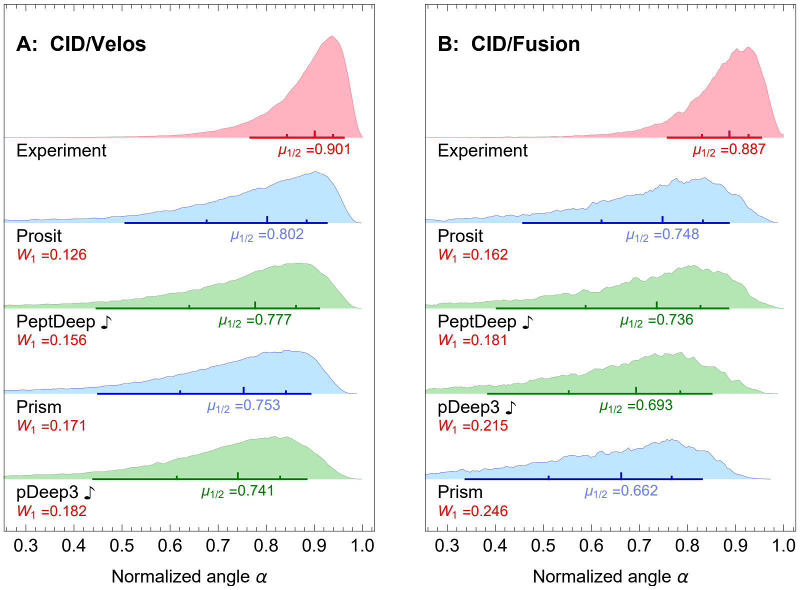

Figure S7: The effect of methionine oxidation (CID; singly-charged). The figure shows the distributions of normalized angle for different methods when applied to peptides with methionine oxidation for CID/Velos (A) and CID/Fusion data (B).
